# Complement induces podocyte pyroptosis in membranous nephropathy by mediating mitochondrial dysfunction

**DOI:** 10.1038/s41419-022-04737-5

**Published:** 2022-03-29

**Authors:** Hui Wang, Daoyuan Lv, Song Jiang, Qing Hou, Lei Zhang, Shen Li, Xiaodong Zhu, Xiaodong Xu, Jianqiang Wen, Caihong Zeng, Mingchao Zhang, Fan Yang, Zhaohong Chen, Chunxia Zheng, Jing Li, Ke Zen, Zhihong Liu, Limin Li

**Affiliations:** 1 National Clinical Research Center of Kidney Diseases, Jinling Hospital, Nanjing University School of Medicine, Nanjing, Jiangsu China; 2 State Key Laboratory of Pharmaceutical Biotechnology, Jiangsu Engineering Research Center for MicroRNA Biology and Biotechnology, Nanjing University School of Life Sciences, Nanjing, Jiangsu China; 3 State Key Laboratory of Natural Medicines, School of Life Science and Technology, China Pharmaceutical University, Nanjing, Jiangsu China

**Keywords:** Cell death, Membranous nephropathy

## Abstract

Podocyte damage mediated by in situ complement activation in the glomeruli is a key factor in the pathogenesis of membranous nephropathy (MN), but the molecular mechanism has not been fully elucidated. Pyroptosis is a special type of programmed cell death, mediate inflammatory response and induce tissue injury. However, it is not clear whether pyroptosis is involved in the development and progression of MN. Here, we report that pyroptosis plays an important role in promoting podocyte injury in MN. We first observed the occurrence of pyroptosis in the kidneys of MN patients and validated that complement stimulation triggered pyroptosis in podocytes and that inhibiting pyroptosis reversed complement-induced podocyte damage in vitro. In addition, stimulation of complement caused mitochondrial depolarization and reactive oxygen species (ROS) production in podocytes, and inhibition of ROS reversed complement-induced pyroptosis in podocytes. Interestingly, inhibition of pyroptosis in turn partially alleviated these effects. Furthermore, we also found the involvement of pyroptosis in the kidneys of passive Heymann nephritis (PHN) rats, and inhibitors of pyroptosis-related molecules relieved PHN-induced kidney damage in vivo. Our findings demonstrate that pyroptosis plays a critical role in complement-induced podocyte damage in MN and mitochondrial dysfunction is an important mechanism underlying this process. It provides new insight that pyroptosis may serve as a novel therapeutic target for MN treatment in future studies.

## Introduction

Membranous nephropathy (MN) is a common cause leading to nephrotic syndrome, and one of the most important reasons for patients with chronic kidney diseases advance to end-stage renal disease (ESRD) [1, 2]. MN is an organ-specific autoimmune disease, and most of MNs are associated with antiphospholipase A2 receptor antibodies, which bind to the corresponding antigens on podocytes to form immune complexes and then activate complement to form C5b-9 membrane attack complexes, which damage podocytes, destroy the glomerular filtration barrier, and produce proteinuria. Therefore, it is widely believed that complement plays a key role of mediation tissue injury in membranous nephropathy, but the molecular mechanism by which complement causes podocyte damage remains unclear.

Pyroptosis is a recently discovered mechanism of programmed cell death [3]. It is a special type of programmed cell death that is characterized by the swelling and rupture of cells, the release of cell contents and a strong inflammatory response. Among these, proinflammatory response are the most distinguishing characteristics between it with apoptosis. The mechanism of pyroptosis occurs through receptors such as Nod-like receptors that recognize endogenous molecules and toxic foreign products of bacteria, viruses and hosts and induce the assembly of inflammasome complexes, thereby stimulating the activation of downstream caspase-1. Activated caspase-1 cleaves gasdermin D (GSDMD) to produce the pore-forming N-terminus of GSDMD (GSDMD-N) to induce pyroptosis. In addition, activated caspase-1 causes the release of the inflammatory factors IL-1β and IL-18, which aggravates the inflammatory response. Pyroptosis has been studied in ischemia-reperfusion injury [4], lupus nephritis [5], diabetic nephropathy [6], HIV-related nephropathy [7], and so on. However, it is not clear whether podocyte pyroptosis is involved in the development and progression of MN.

In this study, we observed that the kidneys tissue of MN patients and PHN rats displayed significant expression of pyroptosis-related molecules. Furthermore, complement stimulation induced pyroptosis of podocytes in vitro, and inhibition of the latter alleviated complement-induced podocyte damage. The flow diagram of the overall experimental idea is shown in Supplementary Fig. S1.

## Results

### Pyroptosis in the kidneys of MN patients

**Fig. 1:**
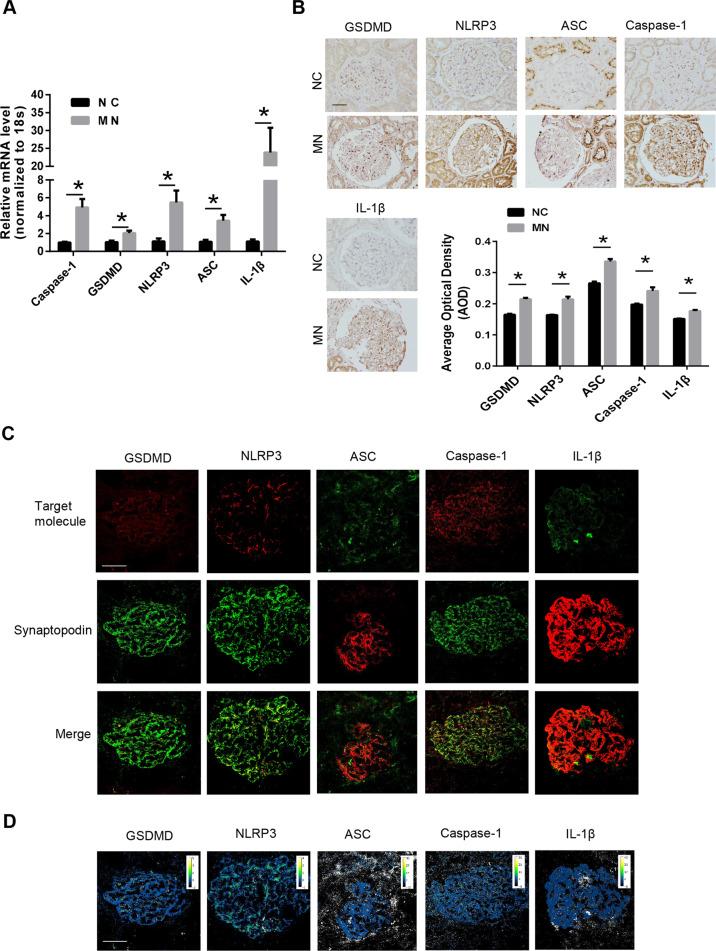
The presence of pyroptosis in the podocytes of MN patients. **A** The relative mRNA levels of pyroptosis-related genes in the glomeruli of healthy controls and MN patients. **B** Immunohistochemical detection of the protein level of pyroptosis-related proteins in renal cortical sections of healthy controls and MN patients (scale bar = 50 μm). The quantification of the average optical density is shown in the panel below (12–81 glomeruli of each group were analyzed). **C** The colocalization of pyroptosis-related proteins and synaptopodin in renal cortical sections of MN patients (scale bar = 50 μm). **D** The ratios of fluorescence intensity of pyroptosis-related proteins to synaptopodin of Panel (**C**) were calculated by the ImageJ program (scale bar = 50 μm). The data represent the mean ± SEM. *p < 0.05.

To determine the existence of pyroptosis in the kidneys of MN patients, the glomeruli of normal controls (NCs) and MN patients isolated by stereomicroscopy were subjected to RNA extraction, reverse transcription, and qRT–PCR assessment. The results showed that the mRNA levels of pyroptosis-related genes, including caspase-1, GSDMD, NLRP3, ASC, and IL-1β, in the glomeruli of MN patients were significantly upregulated (Fig. 1A). Kidney paraffin sections from NCs and MN patients were subsequently used for immunohistochemical staining, and the results showed that the protein levels of pyroptosis-related proteins, including caspase-1, GSDMD, NPRP3, ASC, and IL-1β, in the renal cortex of MN patients were also significantly upregulated (Fig. 1B). To further observe the distribution of pyroptosis-related proteins in the kidney tissue of MN patients, we subsequently performed double-color co-staining of the podocyte marker synaptopodin and these proteins. The results showed the colocalization of podocyte markers and these proteins, indicating the existence of pyroptosis in podocytes (Fig. 1C, D).

### Pyroptosis plays a role in complement-induced podocyte damage in vitro

**Fig. 2:**
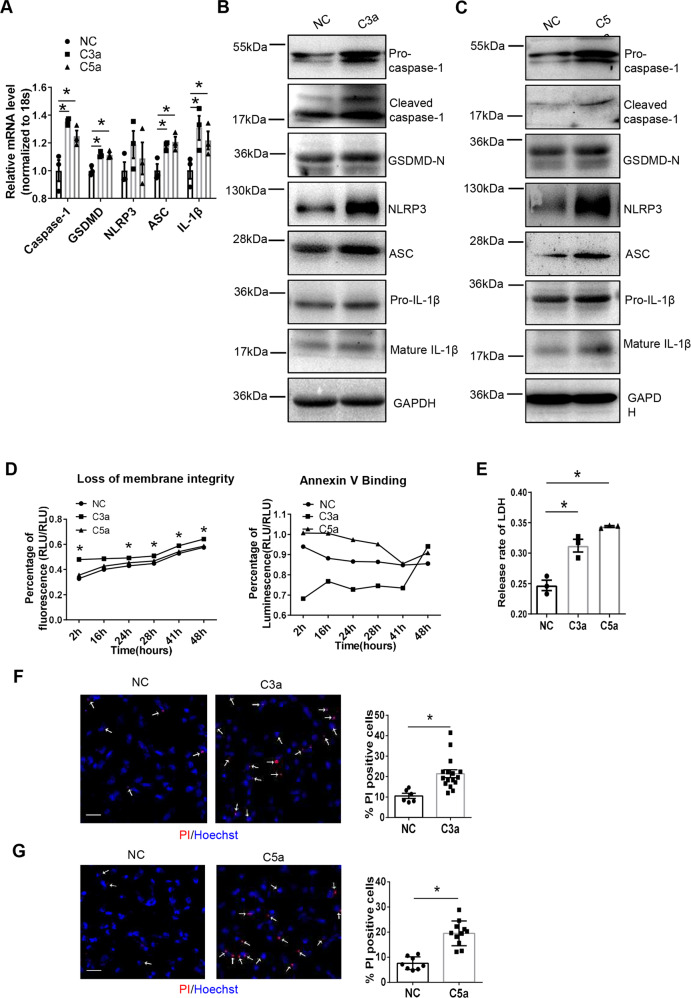
Stimulation of complement triggers podocyte pyroptosis. Podocytes were treated with or without C3a (1 μM) or C5a (100 nM). The pyroptotic features of podocytes, including GSDMD cleavage, membrane integrity, LDH release, and PI uptake, were assessed. **A** The expression of pyroptosis-related mRNAs was measured by qRT–PCR. **B**, **C** The levels of pyroptosis-related proteins were determined by western blotting. **D** The cell membrane integrity and apoptosis of podocytes were assessed using a microplate reader. **E** C3a and C5a promoted the release rate of LDH. **F**, **G** Microscopic imaging of podocytes stained with Hoechst 33342 and propidium iodide (PI) (scale bar = 50 μm). The right panel represents the percentage of PI-positive podocytes (6 and 16 high-power fields were analyzed in group NC and group C3a respectively in (**F**); 8 and 11 high-power fields were analyzed in group NC and group C5a respectively in (**G**)). The data in (**A**), (**D**), and (**E**) represent the mean ± SEM of three independent experiments. *p < 0.05.

Considering the important role of complement in the occurrence and progression of membranous nephropathy, we stimulated podocytes with C3a and C5a in vitro. The results showed that after stimulation of C3a or C5a, the mRNA levels of pyroptosis-related molecules in podocytes were significantly upregulated (Fig. 2A). The Western blotting results showed that stimulation of C3a or C5a significantly increased the protein expression of GSDMD-N, which mediates cell membrane perforation, and other pyroptosis-related proteins (Fig. 2B, C). To verify the damage of complement to podocytes, we further examined cell membrane integrity and apoptosis. The results showed that stimulation of C3a led to the destruction of the cell membrane integrity of podocytes but did not affect podocyte apoptosis over time (Fig. 2D). We also measured the release of LDH to further confirm the permeability of the cell membrane and found that stimulation of C3a and C5a significantly increased the release of LDH from podocytes (Fig. 2E). To assess complement-induced cell death or pyroptosis, human podocytes were cultured with C3a or C5a for 48 h, stained with Hoechst 33342 and PI and examined under a fluorescence microscope. Fig 2F, G shows that the uptake of PI in podocytes was significantly increased after C3a and C5a stimulation. Taken together, the data strongly suggest that C3a and C5a activate pyroptosis in human podocytes.

**Fig. 3:**
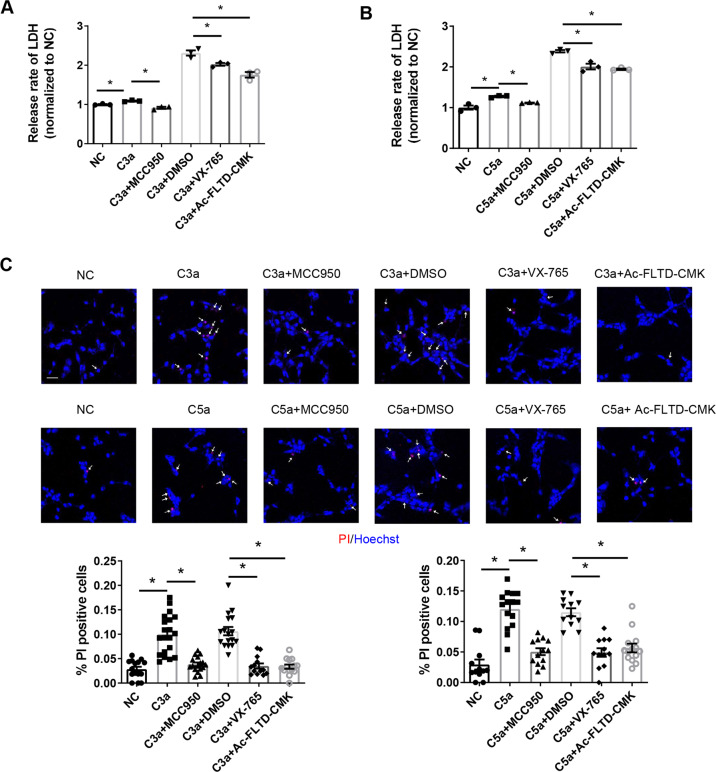
Inhibition of pyroptosis reverses podocyte damage caused by complement stimulation. Podocytes were treated with or without pyroptotic inhibitors under C3a or C5a stimulation conditions. The pyroptotic features of podocytes were determined to validate the reversal effect of pyroptotic inhibitors on complement-induced cell damage. **A**, **B** Release rate of LDH from podocytes. **C** Representative microfluorographs of PI in podocytes cultured with or without pyroptotic inhibitors. Arrows indicate podocytes that stained positive for PI (scale bar = 50 μm). Quantification of PI-positive cells is shown in the panels below (13–20 high-power fields were analyzed in each group in the left panel; 12–15 high-power fields were analyzed in each group in the right panel). The data in (**A**) and (**B**) represent the mean ± SEM of three independent experiments. *p < 0.05.

To elucidate the role of pyroptosis in complement-induced podocyte damage, we incubated podocytes with a series of inhibitors of the key molecules in the process of pyroptosis and assessed LDH release and PI uptake in podocytes. An inhibitor of NLRP3 (MCC950), an inhibitor of caspase-1 (VX-765), and an inhibitor of GSDMD-N (Ac-FLTD-CMK) were used to treat podocytes under C3a or C5a stimulation conditions. After confirming the inhibitory efficiency of each inhibitor (Supplementary Fig. S2), we assessed the release of LDH and the uptake of PI. The results showed that the three inhibitors all significantly reduced complement-induced LDH release (Fig. 3A, B) and PI uptake (Fig. 3C) in podocytes. These data indicate that inhibiting different molecules in the pyroptosis pathway can reverse the damage to podocytes caused by complement.

### Mitochondrial depolarization and ROS production are involved in complement-induced pyroptosis of podocytes, and inhibition of pyroptosis ameliorate above damages

**Fig. 4:**
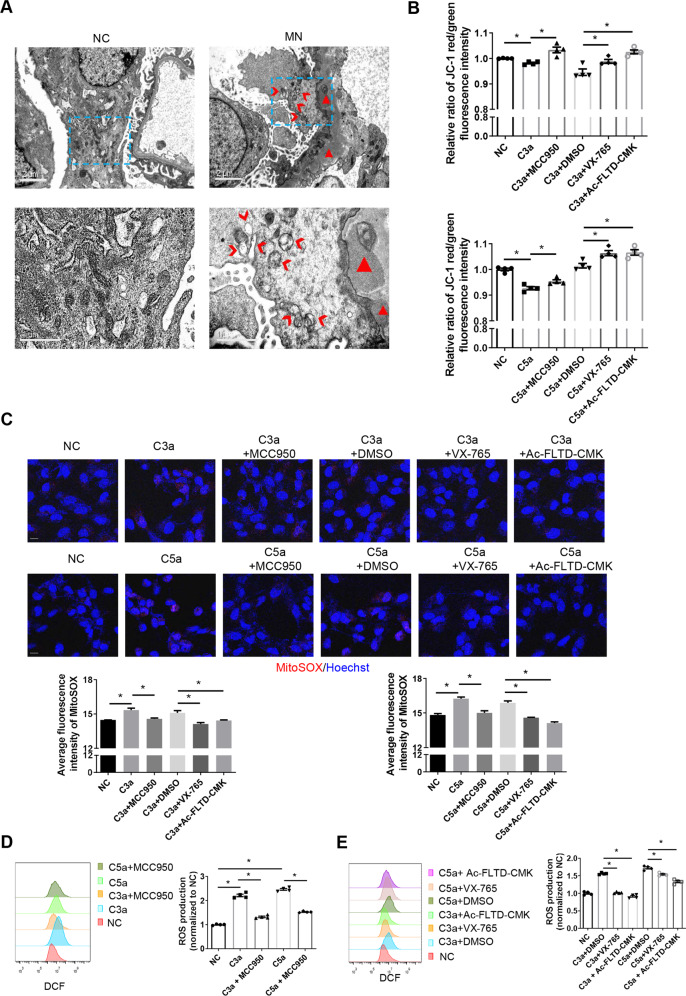
Complement induces the production of ROS and mitochondrial depolarization in podocytes. **A** Electron microscopy observation of changes in mitochondrial morphology in podocytes of healthy controls and MN patients. Triangles (Δ), electron-dense deposits; Arrowheads (>), severe mitochondrial damage: vacuolar degeneration of mitochondria. **B** Relative ratios of JC-1 red/green fluorescence intensity were assessed by flow cytometry to verify the mitochondrial membrane potential in cultured podocytes. The ratio of JC-1 red/green fluorescence reflects the degree of depolarization of the mitochondria. **C**–**E** Inhibitors of pyroptosis-related molecules were used to validate the involvement of ROS in complement-induced pyroptosis. **C** Production of mitochondrial ROS was detected using MitoSOX in cultured podocytes (scale bar = 20 μm). Quantification is shown in the panels below (8–17 high-power fields were analyzed in each group in the left panel; 10–15 high-power fields were analyzed in each group in the right panel). **D**, **E** Production of ROS was detected using flow cytometry in cultured podocytes. Quantification is shown in the right panels. The data in (**B**), (**D**), and (**E**) represent the mean ± SEM of four independent experiments. *p < 0.05.

Electron microscopy observations showed abnormal changes in mitochondrial morphology, such as vacuolar degeneration of mitochondria in podocytes of MN patients (Fig. 4A). Then, we validated the effect of complement stimulation on mitochondrial function in podocytes in vitro. Mitochondrial membrane potential and ROS production were assessed as indicators of mitochondrial function. Flow cytometry was used to detect JC-1 to verify the influence of complement on the mitochondrial membrane potential of podocytes. After complement stimulation, the red/green fluorescence intensity ratio decreased, indicating the enhanced mitochondrial depolarization, while inhibition of pyroptosis could reverse the complement-induced mitochondrial depolarization (Fig. 4B). As shown in Fig. 4C–E, the ROS production detected both by MitoSOX staining (Fig. 4C) and flow cytometry (Fig. 4D, E) is increased in podocytes after complement stimulation. Similarly, inhibition of pyroptosis also reversed the complement-induced ROS production. These results indicate that inhibition of pyroptosis could alleviate the damage to mitochondrial function caused by complement.

### Inhibition of ROS reverses complement-induced pyroptosis of podocytes

**Fig. 5:**
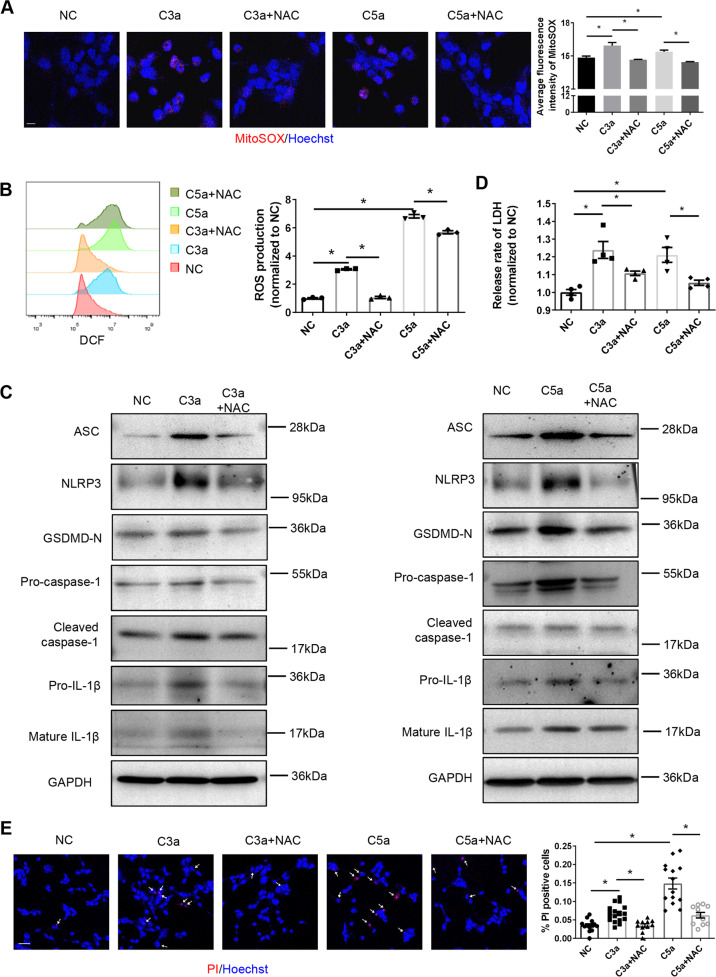
Inhibition of ROS reverses complement-induced pyroptosis of podocytes. An inhibitor of ROS, NAC, was used to demonstrate that the production of mitochondrial ROS mediated C3a- or C5a-induced pyroptosis. **A** Representative images of mitochondrial ROS detected using MitoSOX in cultured podocytes (scale bar = 20 μm). Quantification is shown in the right panel (11–18 high-power fields were analyzed in each group). **B** Production of mitochondrial ROS was detected using flow cytometry in cultured podocytes. Quantification is shown in the right panels. **C** Protein level of pyroptosis-related molecules in podocytes. **D** Release rate of LDH from podocytes. **E** Representative microfluorographs of PI staining in podocytes (scale bar = 50 μm). Quantification is shown in the right panel (11–17 high-power fields were analyzed in each group). The data in (**B**) represent the mean ± SEM of three independent experiments. *p < 0.05. The data in (**D**) represent the mean ± SEM of four independent experiments. *p < 0.05.

We then further explored the role of ROS in complement-induced podocyte pyroptosis. An inhibitor of ROS, acetylcysteine (N-acetylcysteine) (NAC), was used in the experiment. As shown in Fig. 5A, B, NAC significantly inhibited C3a- and C5a-induced ROS production in podocytes. Protein detection results also showed that ROS inhibitors significantly reduced the expression of key protein molecules in the pyroptosis pathway, including ASC, NLRP3, GSDMD-N, pro-caspase-1, cleaved caspase-1, pro-IL-1β, and mature IL-1β (Fig. 5C). Similarly, NAC also significantly reduced the LDH release of podocytes caused by C3a and C5a (Fig. 5D). In addition, PI and Hoechst double overstaining results showed that PI uptake in podocytes induced by C3a and C5a was significantly reduced by NAC (Fig. 5E). All these data suggest that inhibition of ROS can partially reverse complement-induced pyroptosis of podocytes.

### Pyroptosis exists in the kidneys of PHN rats, and inhibition of pyroptosis relieves PHN-induced renal damage in vivo

**Fig. 6:**
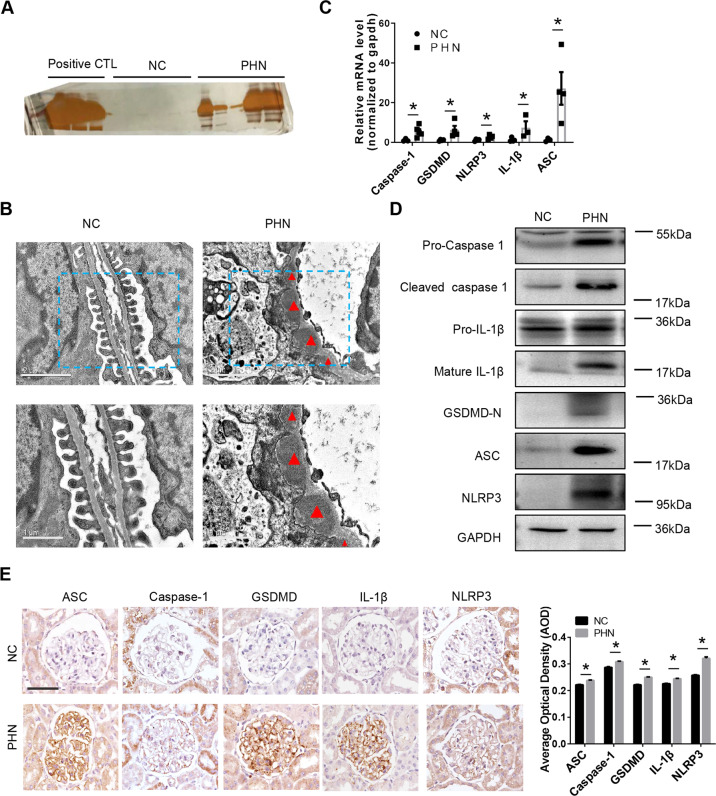
The presence of pyroptosis in the kidney of PHN rats. **A** Silver staining of urine protein of normal control rats and PHN rats. **B** Electron microscopy observation of renal morphology in normal control and PHN rats. Triangles (Δ), electron-dense deposits. **C** Levels of pyroptosis-related mRNAs in glomeruli of normal control (NC) and PHN rats. **D** Western blotting of pyroptosis-related molecules in the renal cortex of normal control rats and PHN rats. **E** Immunohistochemical staining of pyroptosis-related molecules in renal sections of normal control rats and PHN rats (scale bar = 50 μm). Quantification of the average optical density is shown in the right panel (27–256 glomeruli of each group were analyzed). The data in (**C**) and (**E**) represent the mean ± SEM, *p < 0.05.

To test pyroptosis in vivo, we used the PHN rat model to verify the expression of pyroptosis-related molecules. The increase in urinary protein levels (Fig. 6A) and characteristic morphology of MN, such as the subepithelial electron-dense deposits under the electron microscope (Fig. 6B), indicated that the PHN rat model was established successfully. Subsequently, we removed the rat kidneys and separated out the renal cortex within a given time, microseparated the glomeruli, and then purified RNA to assess the expression of pyroptosis-related molecules. The qRT–PCR results showed that compared with the control group, the mRNA levels of pyroptosis-related molecules in the glomeruli of PHN rats were significantly upregulated (Fig. 6C). Western blotting results showed that compared with control rats, the protein levels of pyroptosis-related molecules were significantly upregulated in PHN rats (Fig. 6D). The immunohistochemical staining results also showed upregulation of the protein levels of pyroptosis-related molecules in PHN rats (Fig. 6E).

**Fig. 7:**
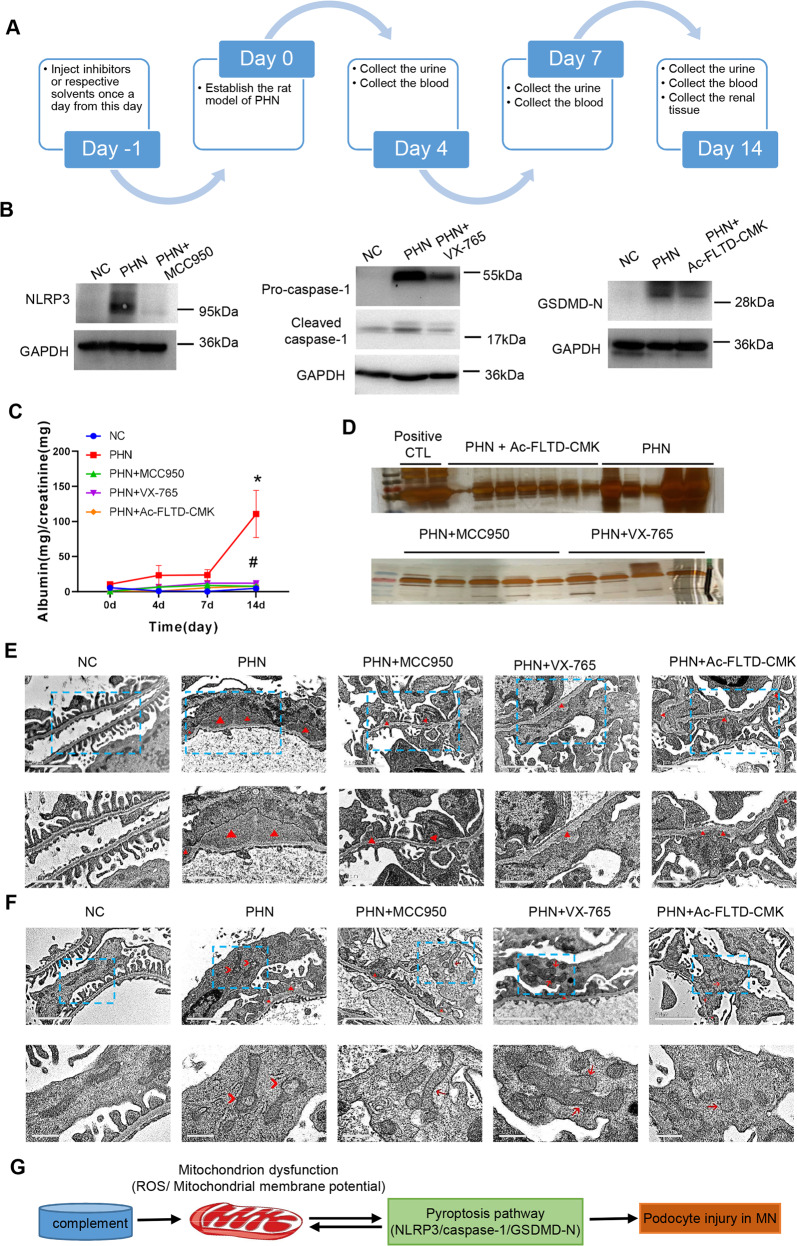
Inhibitors of pyroptosis ameliorate PHN-induced proteinuria and podocyte damage. **A** Schematic diagram of experiment procedure. **B** The efficiency of NLRP3, caspase-1, or GSDMD-N inhibitors in the renal cortex of PHN rats. **C** Albumin-to-creatinine ratio of normal control (NC) and PHN rats with or without inhibitors of pyroptosis. The data represent the mean ± SEM. *p < 0.05, PHN compared with NC at 14d; ^#^*p* < 0.05, PHN + MCC950/ PHN + VX-765/ PHN + Ac-FLTD-CMK compared with PHN at 14d. **D** Silver staining of urine protein of PHN rats with or without inhibitors of pyroptosis. **E** Electron microscopy observation of renal morphology in normal control rats and PHN rats with or without inhibitors of pyroptosis. Triangles (Δ), electron-dense deposits. **F** Electron microscopy observation of mitochondrial morphology in podocytes of normal control rats and PHN rats with or without inhibitors of pyroptosis. Triangles (Δ), electron-dense deposits; Arrows (→), mild mitochondrial dysfunction: mitochondrial cristae disappearance and mitochondrial swelling; Arrowheads (>), severe mitochondrial damage: vacuolar degeneration of mitochondria. **G** Schematic diagram of the molecular mechanism of podocyte pyroptosis induced by complement.

Next, to clarify the role of pyroptosis in the PHN rat model, we used an inhibitor of caspase-1 (VX-765), an inhibitor of NLRP3 (MCC950) and an inhibitor of GSDMD-N (Ac-FLTD-CMK) to treat PHN rats (Fig. 7A). After verifying the suppression efficiency of these inhibitors against the corresponding proteins (Fig. 7B), we tested the proteinuria level of the rats from the normal control group (NC), PHN group (PHN) and inhibitor groups (PHN + VX-765, PHN + MCC950, PHN + Ac-FLTD-CMK). The results showed that compared with the normal control group, the urinary protein of PHN rats was significantly upregulated, and the inhibitors of caspase-1, NLRP3 and GSDMD-N significantly reduced the urinary protein in PHN rats (Fig. 7C). The silver staining method used to verify proteinuria also yielded the same results (Fig. 7D). In addition, the electron microscopy results showed that inhibition of pyroptosis-related molecules partially reduced the subepithelial electron-dense deposits, shown as triangles (Δ), in PHN rats (Fig. 7E). Furthermore, compared with PHN group, all three groups of pyroptosis inhibitors partially alleviated the abnormal changes of mitochondrial morphology such as the less severer of mitochondrial damage and mitochondrial vacuolar degeneration in the podocytes of PHN rats (Fig. 7F). These findings indicate that pyroptosis contributes to renal damage in PHN rats.

## Discussion

Membranous nephropathy is an autoimmune kidney disease, but the mechanisms leading to glomerular damage, especially podocyte damage, remain unclear [8, 9]. Pyroptosis is a special type of programmed cell death [10, 11] and involved in widespread of diseases as well as in kidney diseases, such as acute kidney injury [12], diabetic nephropathy [13], and other chronic progressive diseases [5]. In the present study, we demonstrated pyroptosis occurred in the podocytes of patients with MN and a PHN rat model by showing the significant expression of pyroptosis-related molecules, such as caspase-1, GSDMD-N, NPRP3, ASC, and IL-1β. In addition, we used inhibitors of caspase-1, NLRP3 and GSDMD-N, verifying that inhibition of the pyroptosis process could significantly mitigate proteinuria and kidney damage in PHN rats, indicating the potential for the treatment of MN with inhibitors of Caspase-1, NLRP3, and GSDMD-N. Today, the treatment of MN mainly dependent on immunosuppressive therapy, including cyclophosphamide, calcineurin inhibitors and anti-CD20 antibody (rituximab), our findings provide a new way to explore more comprehensive treatment for patients with MN in the future.

A large body of evidence points to a central role of complement in the pathogenesis of MN [14, 15]. Calcium influx and tyrosine kinase receptor transactivation [16], GTPase and RohA protein activation [17], phospholipid hydrolysis and prostacyclin production [18], apoptosis signal-regulated protein kinase [15], DNA damage [19], endoplasmic reticulum stress, autophagy [20], etc., are involved in the process of complement-mediated podocyte injury. Recent studies suggested the link between complement and pyroptosis events [21, 22]. In the present study, we found that complement can induced podocyte pyroptosis through mediating mitochondrial dysfunction, and revealed an novel mechanism of podocyte injury in MN. The in vitro study showed that complement can induce pyroptosis of podocytes, which was manifested by the increased levels of pyroptosis-related molecules, the loss of cell membrane integrity, the increased release of LDH and the increased staining of PI. Inhibitors of caspase-1, NLRP3, and GSDMD-N could reverse the damage to podocytes caused by complement. The in vivo results indicated that the increased expression of caspase-1, GSDMD-N, NPRP3, ASC and IL-1β in the renal cortex of PHN rats provides strong evidence of pyroptosis participate in the renal damage in MN.

It is well known that excessive levels of ROS causes damage to DNA, proteins or lipids, leading to apoptotic or necroptotic cell death [23–28]. Recent studies have shown that ROS can play a key role in regulating pyroptosis at different levels, including allowing the priming and activation of the NLRP3 inflammasome and the cleavage of GSDMD [29–31]. One of the main sources of ROS are mitochondrial ROS (mtROS) [32]. It was reported that mitochondrial dysfunction and mtROS overexpression can lead to NLRP3 inflammasome activation [33]. Thus, a disrupted mitochondrial membrane potential and ROS generation are tightly associated with pyroptosis. In addition, the production of ROS is also an indicator of podocyte injury attacked by complement [34]. Thus, it is interesting to ask whether complement can induce pyroptosis of podocytes by mediating mitochondrial dysfunction and ROS production. Our mechanistic study revealed that complement-induced podocyte pyroptosis accompanied by mitochondrial depolarization and ROS production, and these effects reversed by inhibition of ROS, indicating a correlation between pyroptosis and mitochondrial dysfunction (Fig. 7G). This finding may also be used to interpret the previous report that the beneficial effects of antioxidants and oxygen free radical scavengers used in MN animal models [35]. Further studies are needed to explore the molecular network in the process of complement-mediated podocyte injury.

In conclusion, Our findings demonstrate that pyroptosis plays a critical role in complement-induced podocyte damage in MN and mitochondrial dysfunction is an important mechanism underlying this process. It provides new insight that pyroptosis may serve as a novel therapeutic target for MN treatment in future studies.

## Materials and methods

### Patient kidney tissues

The kidney tissues of the patients came from the Renal Biobank of National Clinical Research Center of Kidney Diseases, Jiangsu Biobank of Clinical Resources. The inclusion criteria for patients with MN were as follows: (1) primary MN diagnosed by renal biopsy, excluding secondary and other kidney diseases; (2) serum PLA2R antibodies and renal tissue PLA2R staining were double negative or double positive; and (3) IgG subtype staining results showing that IgG4 was the main subtype. The exclusion criteria were as follows: (1) patients with hepatitis B, other autoimmune diseases, other glomerular diseases or interstitial diseases of the interstitial organs; (2) patients without kidney tissue specimens; and (3) patients whose crescent formation or glomerular infiltration cells could be seen under a light microscope. The numbers of patients in goup NC and MN are 4 and 9, respectively.

### Rat models

The SD rats needed for these experiments were female, purchased from Shanghai Sippr-BK Laboratory Animal Corp. Ltd, weighing (170 ± 20) g. The rats were bred in the Department of Comparative Medicine of Jinling Hospital. The animal experiments in this study all followed the norms and guidelines of the Animal Ethics Committee of Jinling Hospital. The PHN rat model was established as previously described [36]. For inhibition of pyroptosis in vivo, PHN rats were administered MCC950 sodium (MCE, New Jersey, USA, HY-12815A, intraperitoneal injection, 10 mg/kg), belnacasan (VX-765) (MCE, New Jersey, USA, HY-13205, gavage, 100 mg/kg) or Ac-FLTD-CMK (MCE, New Jersey, USA, HY-111675, intraperitoneal injection, 2.5 mg/kg) once a day [37–39]. Totally, the mice were randomly divided into five groups, which were not blinded to investigators. The numbers of rats are 5, 5, 5, 5, 7 in group NC, PHN, PHN + MCC950, PHN + VX-765, PHN + Ac-FLTD-CMK, respectively.

### Cell culture and treatment

Conditionally immortalized human podocytes donated by Professor Saleem of Bristol University were cultured and differentiated as described previously [40]. For cell differentiation, podocytes were grown at 37 °C. After differentiation, podocytes were stimulated with 1 μM C3a (Novoprotein, Shanghai, China, # CP21), 100 nM C5a (Novoprotein, Shanghai, China, # CR52), MCC950 sodium (10 μM), belnacasan (VX-765, 20 μM), Ac-FLTD-CMK (10 μM), or NAC (Selleckchem, Houston, Texas, USA, S1623, 10 μM).

### RNA extraction and Real-time PCR

Fresh patient kidney tissue was quickly put into RNAlater^TM^ Stabilization Solution (Thermo Fisher Scientific, Waltham, MA, USA, AM7021) and stored in a −80 °C freezer. To obtain glomerular tissue, we peeled off the glomerulus under a stereomicroscope. Glomerular samples were taken for RNA extraction using an RNeasy Mini Kit (Qiagen, Cat No. 74104). Cell samples were taken for RNA extraction using TRIzol (Invitrogen, 15596–026). The PrimeScript^TM^ RT Master Mix kit (Takara RR036A) was used for reverse transcription. The TB Green® Premix Ex Taq^TM^ II (Tli RNaseH Plus) kit was used for qRT–PCR. Gene expression was normalized to 18 S rRNA or GAPDH for human or rat genes, respectively. Primers used in qRT–PCR were listed in Supplementary Table S1.

### Western blotting

Protein was extracted with RIPA buffer, and a BCA kit (Beyotime, P0012) was used to determine the protein concentration. Experiments were performed as previously described [41]. Primary antibodies against GSDMD-N (Abcam, Cambridge, UK, ab215203), NLRP3 (Proteintech, Chicago, Illinois, USA, 19771–1-AP), ASC(Santa Cruz, Heidelberg, Germany, sc-514414), caspase-1 (Proteintech, Chicago, Illinois, USA,22915-1-AP), GSDMD-N(Affinity Bioscience, AF4012) and IL-1β (Cell Signaling Technology, Danvers, Massachusetts, USA, #12242) and secondary antibodies against mouse and rabbit immunoglobulin were used for detection. GAPDH (Proteintech, Chicago, Illinois, USA, HRP-60004) were used as internal controls.

### Detection of cell membrane integrity and apoptosis

The assay was carried out following the instructions of the RealTime-Glo^TM^ Annexin V Apoptosis and Necrosis Assay (Promega, JA1011). Briefly, podocytes were seeded in a sterile 96-well cell culture plate. The 2× detection reagents were prepared and added to the wells. The plate was shaken at a speed of 500–700 rpm for 30 s. Then, we incubated the cells at 37 °C and used a microplate reader to detect the luminescence and fluorescence values of each well after different time periods.

### Lactate dehydrogenase detection

A lactate dehydrogenase cytotoxicity detection kit (Beyotime, C0016) was used for detection. We collected the cell culture medium for assessment according to the manufacturer’s instructions [42]. Briefly, 1 h before the scheduled assessment point, we added one-tenth of the volume of the culture solution to the LDH release reagent in the sample’s maximum enzyme activity hole, pipetting and mixing repeatedly, and placed it in a 37 °C incubator. And, 1 h later, we centrifuged the cell culture plate at 400 g for 5 min at room temperature. A total of 120 μl of cell supernatant was taken from each well and added to a new 96-well plate for testing. Freshly prepared LDH detection working solution was added at 60 μl/well, followed by mixing and incubation at room temperature for 30 min in the dark. Then, a microplate reader was used to detect the absorbance at 490 nm, and 600 nm was used as the reference wavelength.

### Detection of urine protein and creatinine in rats

Urine protein was detected by the CBB method with a Urine Protein Test Kit (Nanjing Jiancheng, C035–2-1) and by silver staining with a Fast Silver Stain Kit (Beyotime, P0017S). The creatine oxidase method with a Creatinine Assay Kit (Nanjing Jiancheng, C011–1-1) was used to detect creatinine.

### Mitochondrial membrane potential detection

A MitoProbe^TM^ JC-1 Assay Kit (Thermo Fisher Scientific, M34152) was used for membrane potential detection [43]. Briefly, cells were resuspended in 1 ml phosphate buffered saline (PBS) at approximately 1 × 10^6^ cells/ml. Wells containing 1 μl of 50 mM CCCP were set as positive wells and incubated at 37 °C for 5 min. Then, we added 10 µl of 200 μM JC-1 to each well and incubated the cells at 37 °C for 30 min. Finally, the cells were washed twice with PBS and resuspended for flow cytometry detection.

### Active oxygen detection

A MitoSOX^TM^ Red mitochondrial superoxide indicator kit (Thermo Fisher Scientific, M36008) and Reactive Oxygen Species Assay Kit(Beyotime, S0033M) was used for detection of active oxygen [44]. For MitoSOX^TM^ Red mitochondrial superoxide indicator kit, after preparing the 5 μM working solution, we added 1 ml working solution to cells of each sample well and incubated cells at 37 °C for 10 min in the dark. Cells were then washed with PBS and stained with Hoechst. Confocal microscope was used for fluorescence imaging. For Reactive Oxygen Species Assay Kit, we added 1 ml diluted DCFH-DA to cells of each sample well and incubated cells at 37 °C for 20 min in the dark. Cells were collected and detected by flow cytometry.

### Immunohistochemistry staining

Paraffin tissue sections were dewaxed with different concentrations of xylene, ethanol, and water in turn. Then, antigen retrieval was performed. After blocking the slices with 10% calf serum for 30 min, we incubated them overnight with primary antibodies at 4 °C. Secondary antibodies were incubated at room temperature for 30 min. DAB was used to develop color, and hematoxylin was used to stain the nucleus [45].

### Ultrastructural study by transmission electron microscopy

Scanning electron microscopy was performed as previously described [46]. We focused on subepithelial electron-dense deposits and morphological changes in the mitochondria in the podocytes.

### Statistical analysis

All experiments were carried out at least three times in triplicate. All the statistical tests were justified as appropriate. Analysis of variance was performed and assumption criteria were met and analysis of variance was performed. All of the data are presented as the mean ± SEM. Statistical analysis was performed using PRISM 8 (GraphPad Software Inc, USA). Comparisons of quantitative data between two groups were analyzed with a *t*-test (two-tailed; *p < 0.05 was considered significant).

### Study approval

Studies using human samples were carried out in accordance with approved guidelines from the local committee on human subjects at Jinling Hospital, Nanjing University School of Medicine, China (2013KLY-012). All participants provided written informed consent for participation. All animal protocols and procedures were approved by the Institutional Animal Care and Use Committee at Jinling Hospital.

## Supplementary information

Supplementary Figure Legends

Supplementary Figure S1

Supplementary Figure S2

Supplementary Table S1

Full scans of uncropped blots

checklist

## Data Availability

The data supporting the findings of this study are available in the manuscript or supplementary materials. Additional data are available from the corresponding authors upon reasonable request.
