# Supplementary material for: Complement induces podocyte pyroptosis in membranous nephropathy by mediating mitochondrial dysfunction
Source: Cell Death Dis. 2022 Mar 29;13(3):281. doi: 10.1038/s41419-022-04737-5 (PMC8964685; doi:10.1038/s41419-022-04737-5)
Supplement: Supplementary file 1 — Supplementary Figure Legends [file 41419_2022_4737_MOESM1_ESM.docx]

Supplementary Information

**Complement induces podocyte pyroptosis in membranous nephropathy by mediating mitochondrial dysfunction**

Hui Wang^1^, Daoyuan Lv^1^, Song Jiang^1^, Qing Hou^1^, Lei Zhang^2^, Shen Li^1^,Xiaodong Zhu^1^, Xiaodong Xu^1^, Jianqiang Wen^1^, Caihong Zeng^1^, Mingchao Zhang^1^, Fan Yang^1^, Zhaohong Chen^1^, Chunxia Zheng^1^, Jing Li^2^, Ke Zen^2,3^, Zhihong Liu^1¶^, and Limin Li^3¶^

From ^1^ National Clinical Research Center of Kidney Diseases, Jinling Hospital, Nanjing University School of Medicine, Nanjing, Jiangsu, China; ^2^State Key Laboratory of Pharmaceutical Biotechnology, Jiangsu Engineering Research Center for MicroRNA Biology and Biotechnology, Nanjing University School of Life Sciences, Nanjing, Jiangsu, China; ^3^State Key Laboratory of Natural Medicines, School of Life Science and Technology, China Pharmaceutical University, Nanjing, Jiangsu, China.

**Supplementary Figure S1. The flow diagram of the overall experimental idea.**

This study were carried out from three levels of MN patients, rats and cells. Firstly, the existence of pyroptosis in the kidney of MN patients was verified by qRT-PCR and immunohistochemistry. Subsequently, qRT-PCR, western blot, membrane integrity, LDH, and PI were used to verify the effect of complement stimulation on podocyte pyroptosis *in vitro*. The changes of LDH and PI were detected after the use of pyroptosis-related molecular inhibitors to verify the effect of pyroptosis on complement-induced podocyte injury. The role of mitochondria in pyroptosis was subsequently verified. The changes of mitochondria in podocytes of MN patients were observed by electron microscope. Levels of JC-1 and ROS were used to verify the effect of complement on podocyte mitochondria and the role of pyroptosis on complement induced podocyte injury. ROS staining, western blot, LDH and PI were used to verify the effect of ROS inhibitors on complement-induced podocyte pyroptosis. Finally, pyroptosis was verified in the PHN rat model, and podocyte damage was observed by urine protein detection and electron microscope. The existence of pyroptosis in the kidney was verified by qRT-PCR, western blot and immunohistochemistry. The effect of pyroptosis on renal injury in PHN rats was then verified using pyroptosis inhibitors.

**Supplementary Figure S2. Inhibition efficiency of pyroptosis inhibitors at the cellular level.**

Western blot were used to detect the protein level of NLRP3, caspase-1 or GSDMD-N in podocytes cultured in vitro with the presence of C3a, with or without inhibitors of these particular pyroptosis-related molecules, in order to verify the efficiency of the inhibitors.
