# Supplementary figures and images for: Complement induces podocyte pyroptosis in membranous nephropathy by mediating mitochondrial dysfunction
Source: Cell Death Dis. 2022 Mar 29;13(3):281. doi: 10.1038/s41419-022-04737-5 (PMC8964685; doi:10.1038/s41419-022-04737-5)

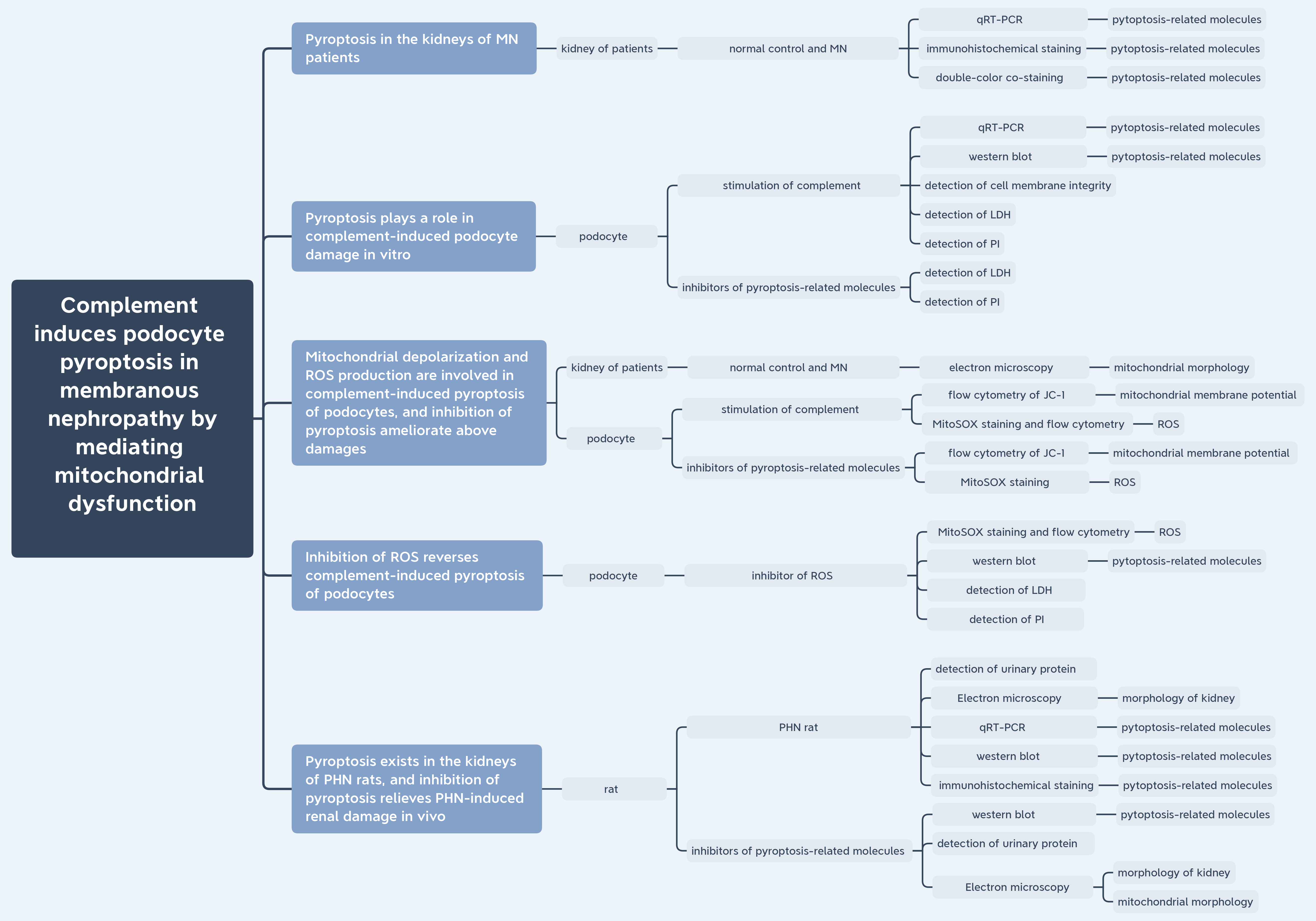

Supplement: Supplementary file 2 — Supplementary Figure S1 [file 41419_2022_4737_MOESM2_ESM.tif]

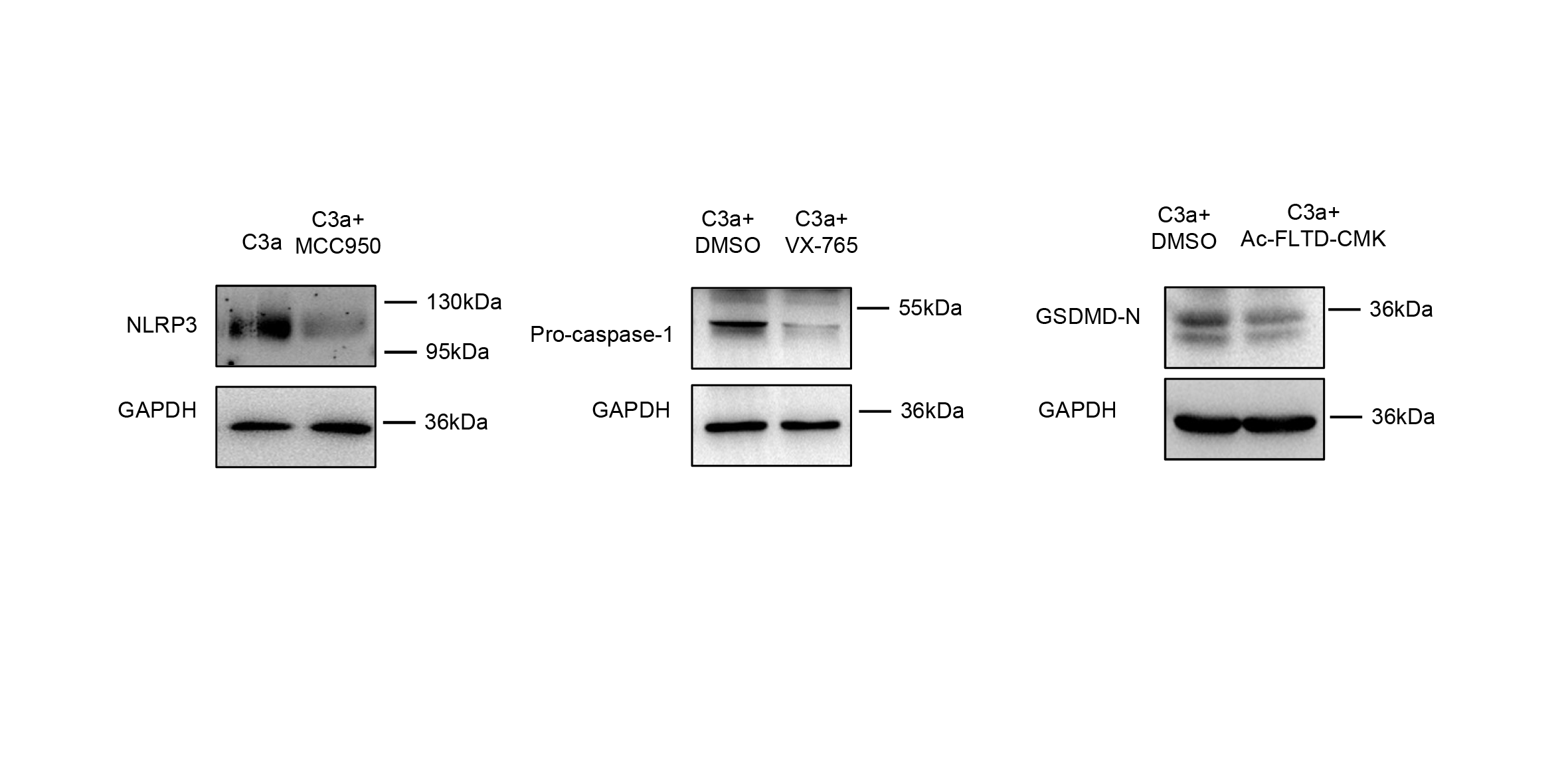

Supplement: Supplementary file 3 — Supplementary Figure S2 [file 41419_2022_4737_MOESM3_ESM.tif]
