# Supplementary material for: Complement induces podocyte pyroptosis in membranous nephropathy by mediating mitochondrial dysfunction
Source: Cell Death Dis. 2022 Mar 29;13(3):281. doi: 10.1038/s41419-022-04737-5 (PMC8964685; doi:10.1038/s41419-022-04737-5)
Supplement: Supplementary file 4 — Supplementary Table S1 [file 41419_2022_4737_MOESM4_ESM.docx]

**Supplementary Table S1. Primers used in qRT-PCR**

| gene | forward | reverse |
| --- | --- | --- |
| *18S* (human) | TTTCTCGATTCCGTGGGTGG | AGCATGCCAGAGTCTCGTTC |
| *CASP1* (human) | AGTCGGCAGAGATTTATCCA | GTACCCCAGATTTTGTAGCAG |
| *GSDMD* (human) | GAAGAAGACGGTCACCATCCCC | GGTCCTCTGCTTCTTATCCGGGAA |
| *NLRP3* (human) | GGTTCAGATAATGCACGTGTT | ATTCCTGTCTTCAATGCACT |
| *IL1B* (human) | AATGACAAAATACCTGTGGCCTTG | TGGGCAGACTCAAATTCCAG |
| *ASC* (human) | CTCCTCAGTCGGCAGCCAAG | CAAGTCCTTGCAGGTCCAGT |
| *Casp1* (rat) | GACTCTAGACTACAGATGCCAAC | CTTCTTATTGGCATGATTCCC |
| *Gsdmd* (rat) | CTCCCTGGCCCTATTGTCA | GTCTTGCTGCATTCTAACCCTG |
| *Nlrp3* (rat) | CCCTGAGATGTTTCAATATTAGCA | AGGAATGAAAACTCCGTGTGA |
| *Il1b* (rat) | TAAAGATGGCTGCACTATTCCT | ACACACGTTTTCTTTGACCA |
| *Asc* (rat) | CCCATAGACCTCACTGATAAACT | GCTCCAGACTCTTCCATAATCTT |
| *Gapdh* (rat) | TCCCTCAAGATTGTCAGCAA | AGATCCACAACGGATACATT |
