# Supplementary material for: Complement induces podocyte pyroptosis in membranous nephropathy by mediating mitochondrial dysfunction
Source: Cell Death Dis. 2022 Mar 29;13(3):281. doi: 10.1038/s41419-022-04737-5 (PMC8964685; doi:10.1038/s41419-022-04737-5)

Figure 2B

pro-caspase-1

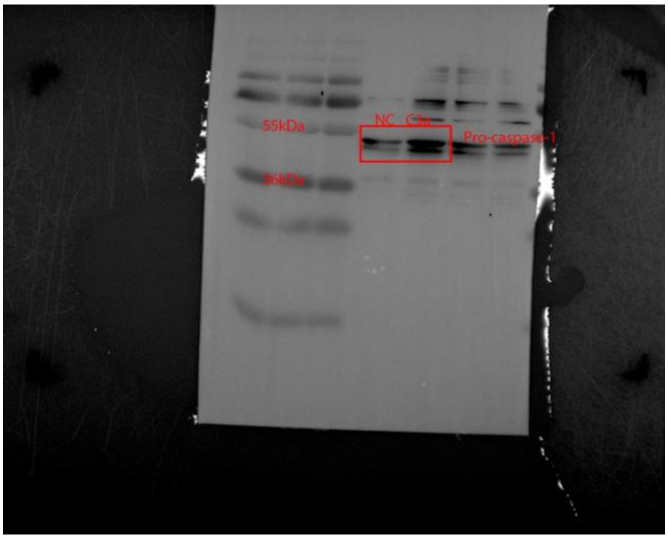

cleaved caspase-1

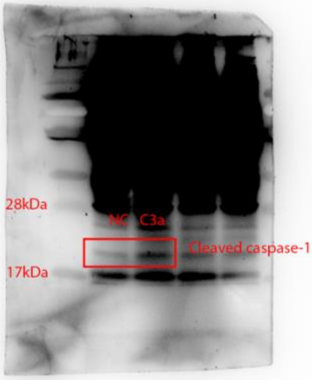

GSDMD-N

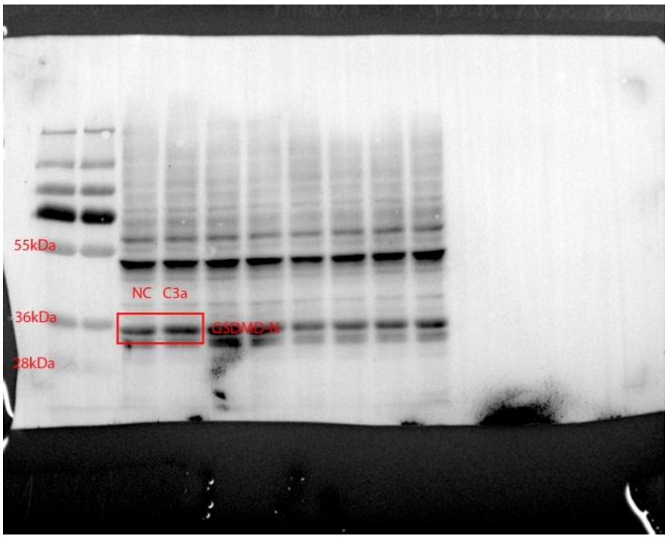

NLRP3

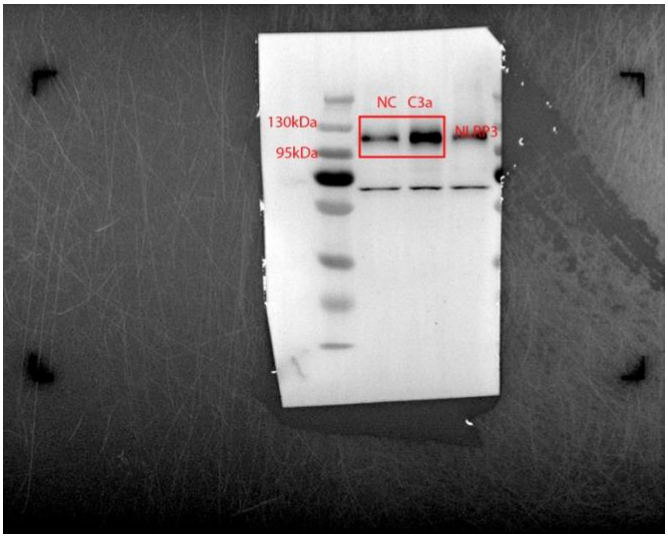

ASC

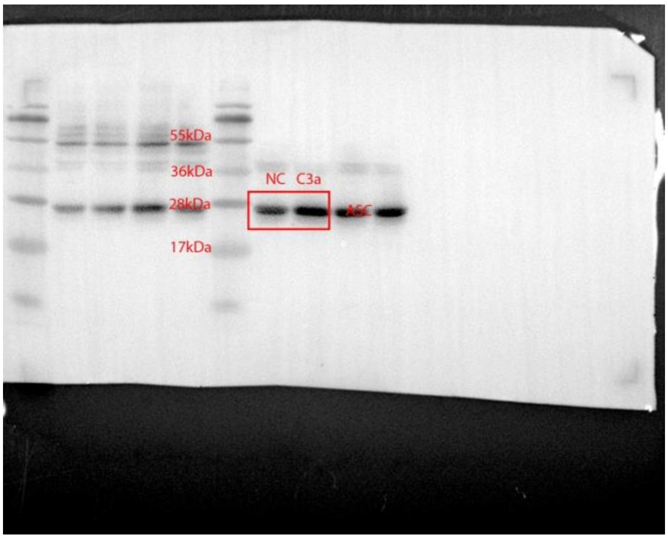

pro-IL-1β

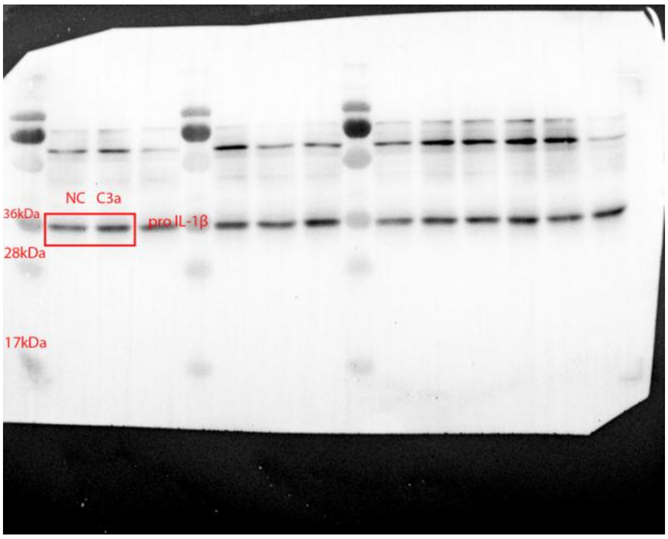

mature IL-1β

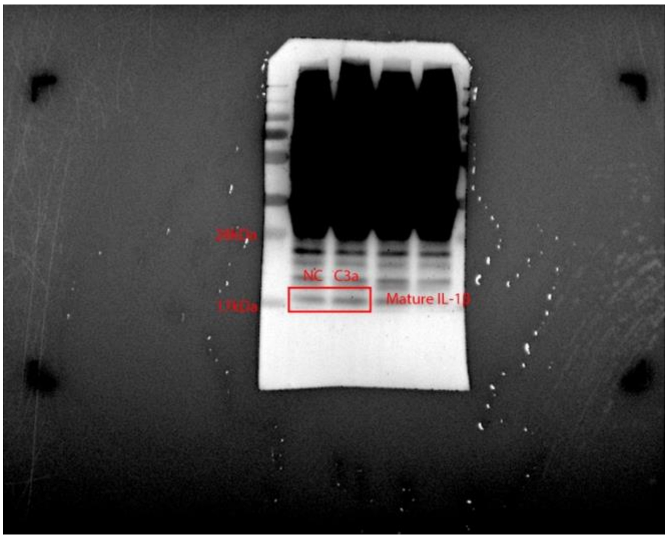

GAPDH

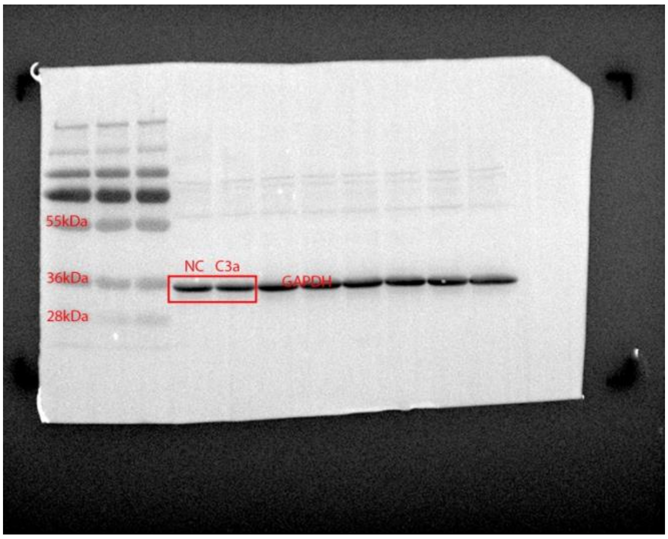

Figure 2C

pro-caspase-1

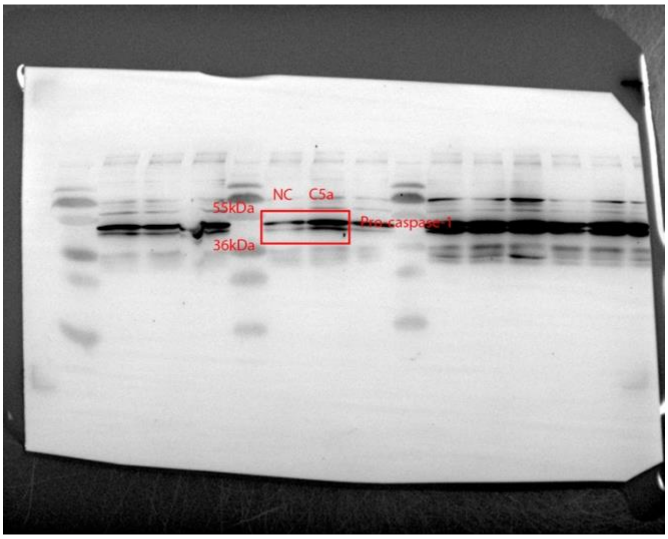

cleaved caspase-1

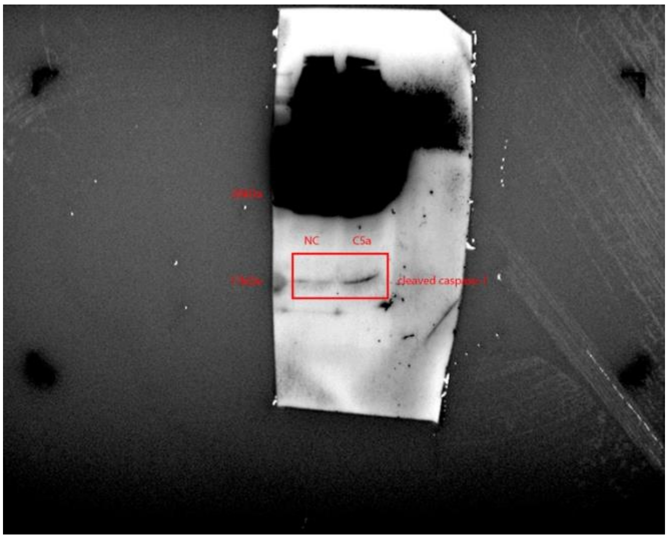

GSDMD-N

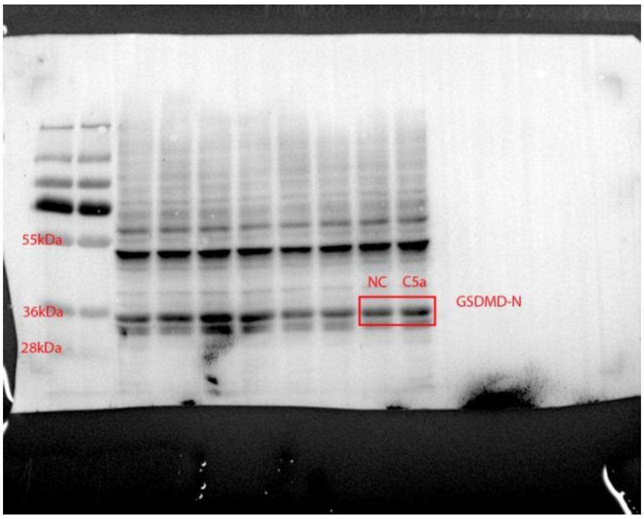

NLRP3

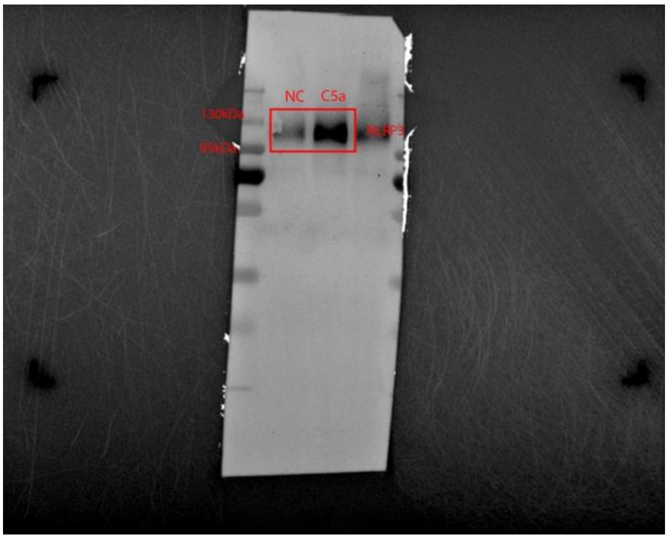

ASC

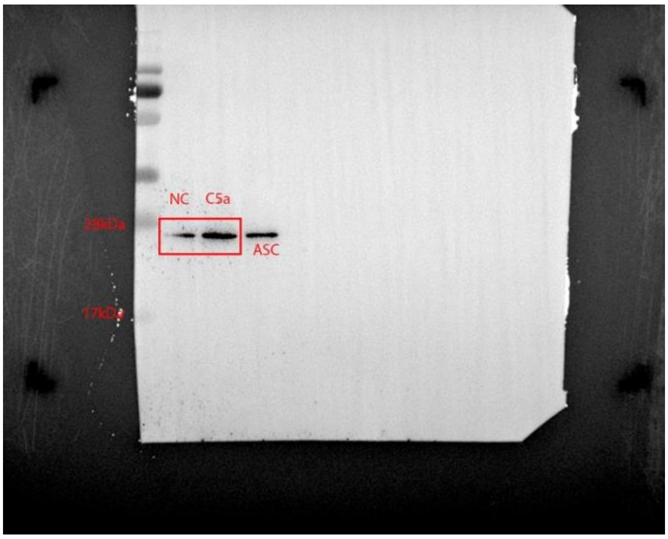

pro-IL-1β

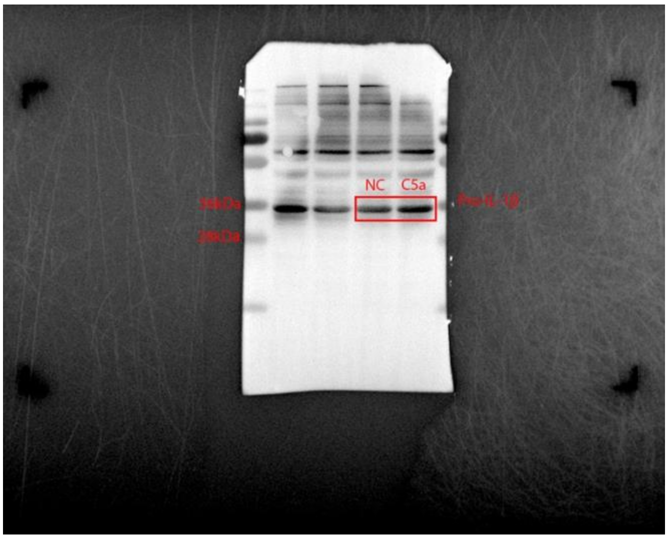

mature IL-1β

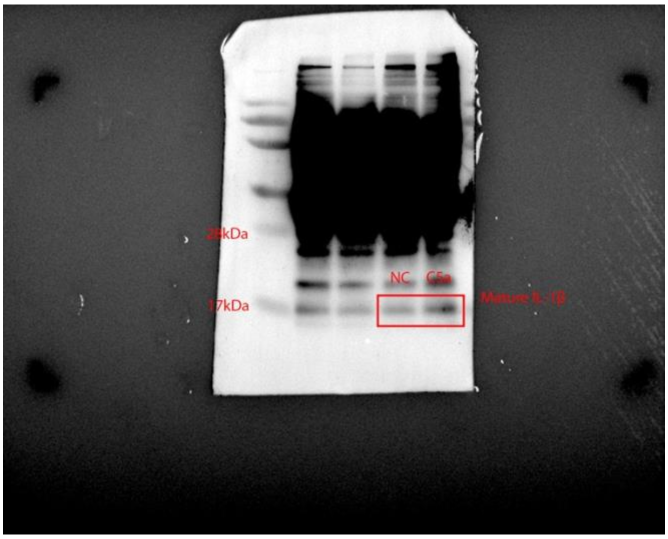

GAPDH

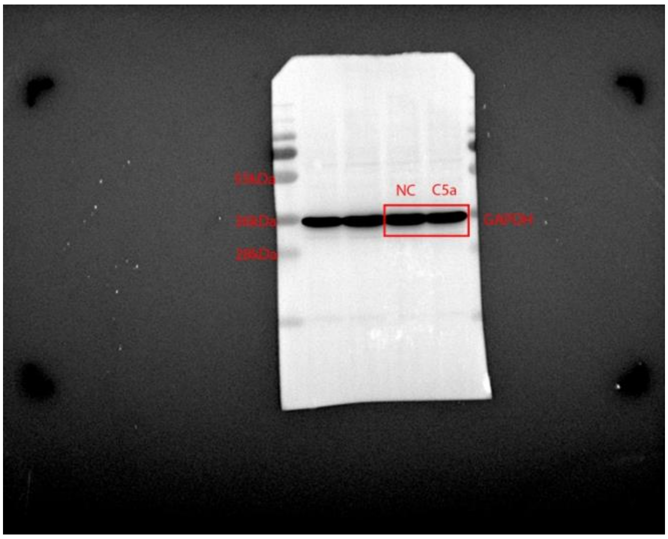

left of Figure 5C

ASC

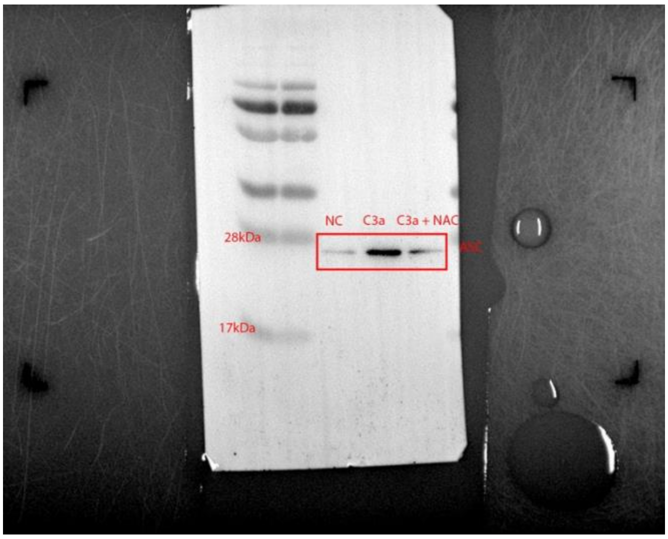

NLRP3

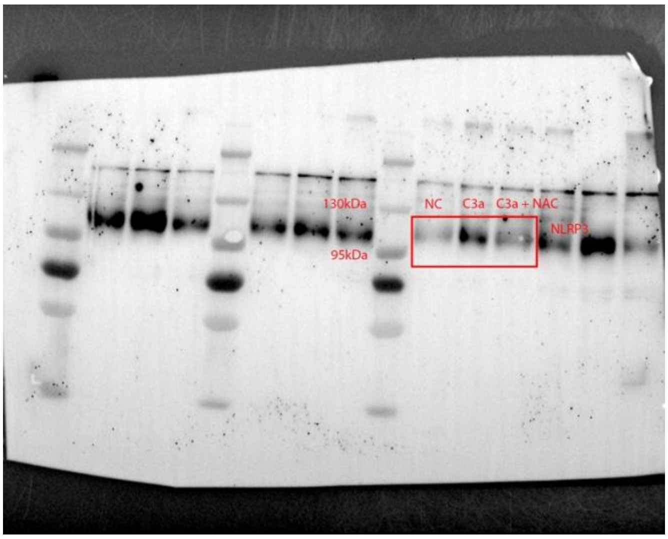

GSDMD-N

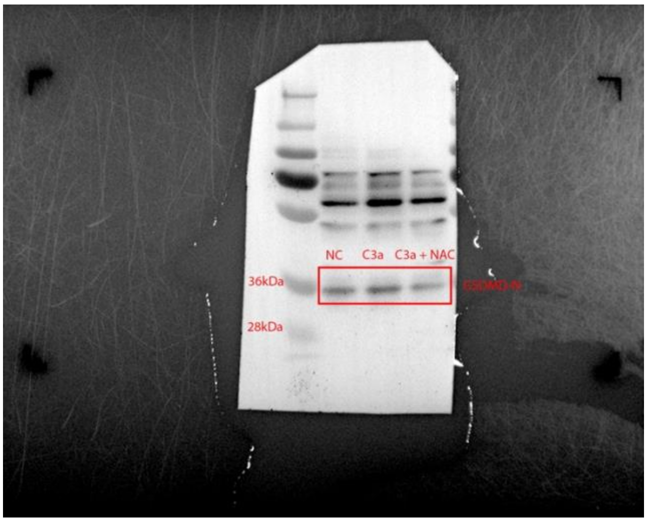

pro-caspase-1

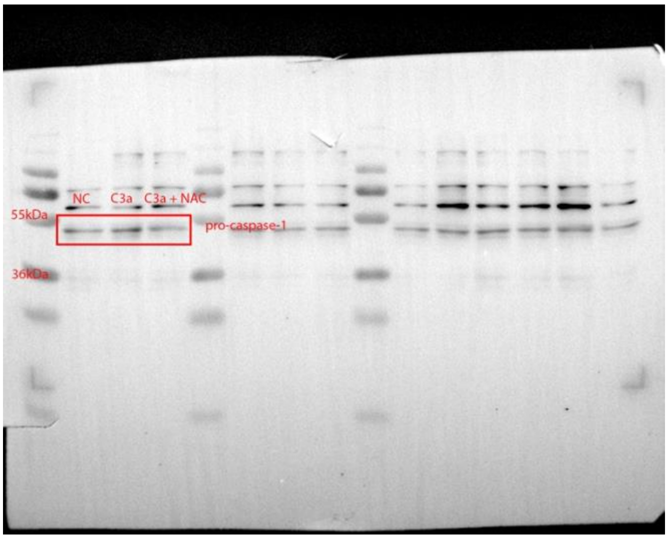

cleaved caspase-1

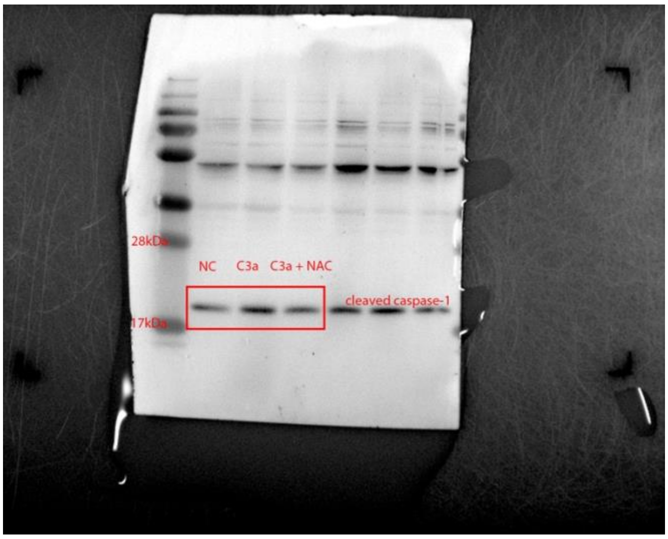

pro-IL-1β

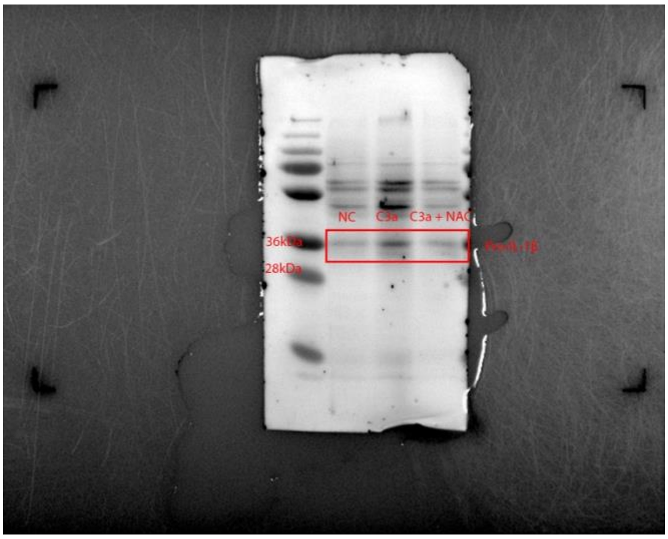

mature IL-1β

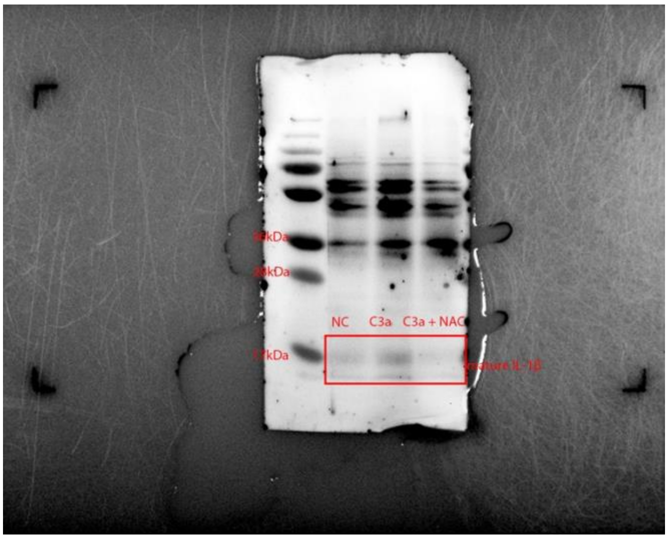

GAPDH

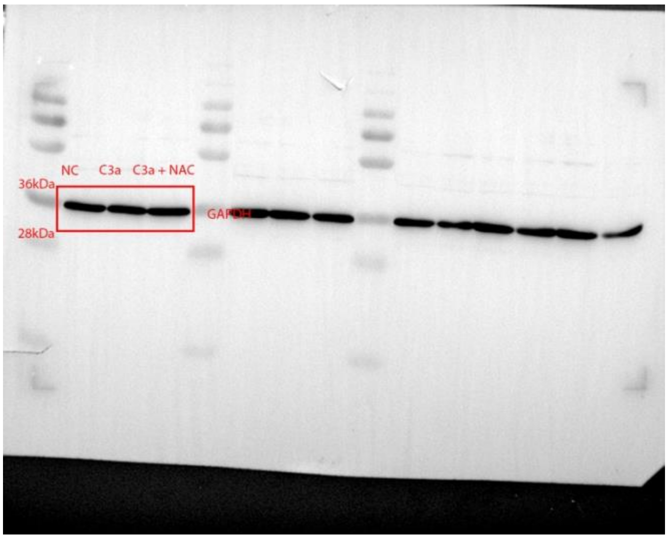

right of Figure 5C

ASC

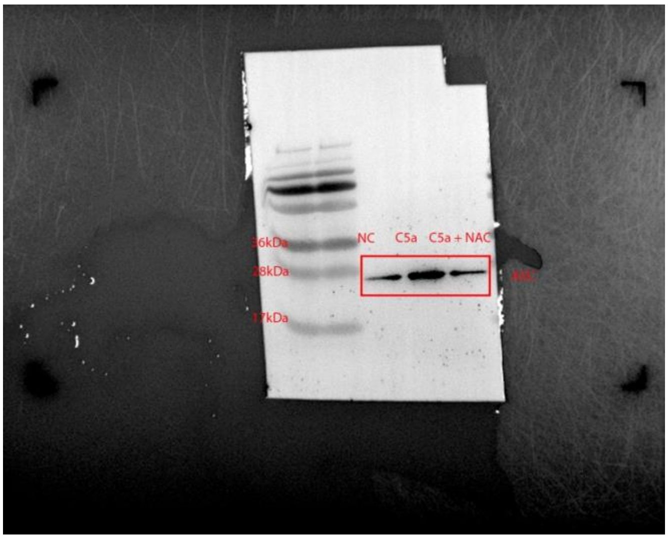

NLRP3

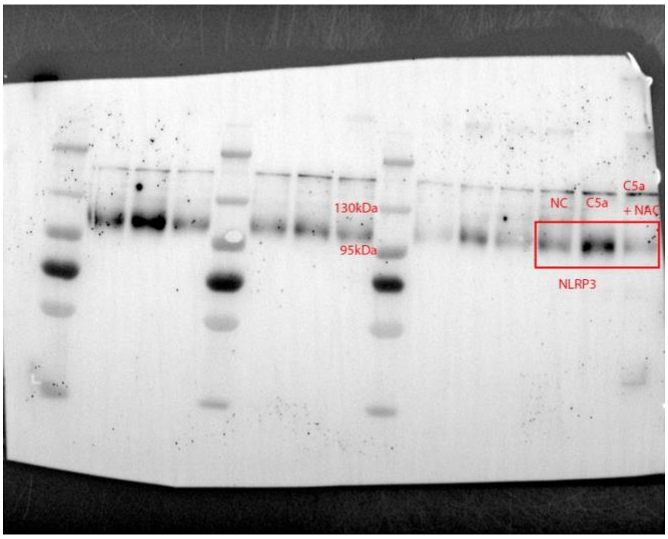

GSDMD-N

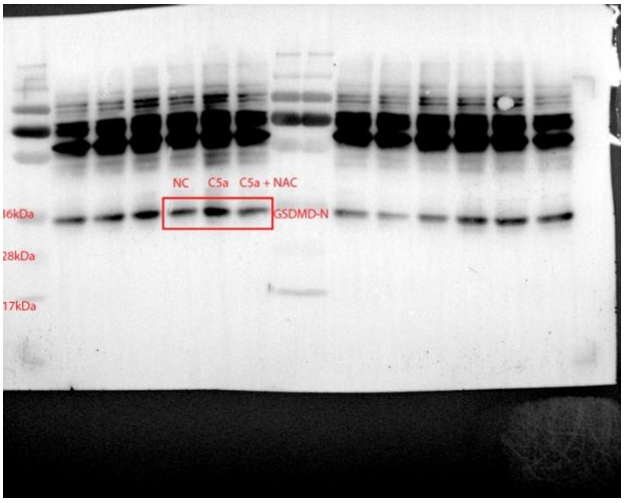

pro-caspase-1

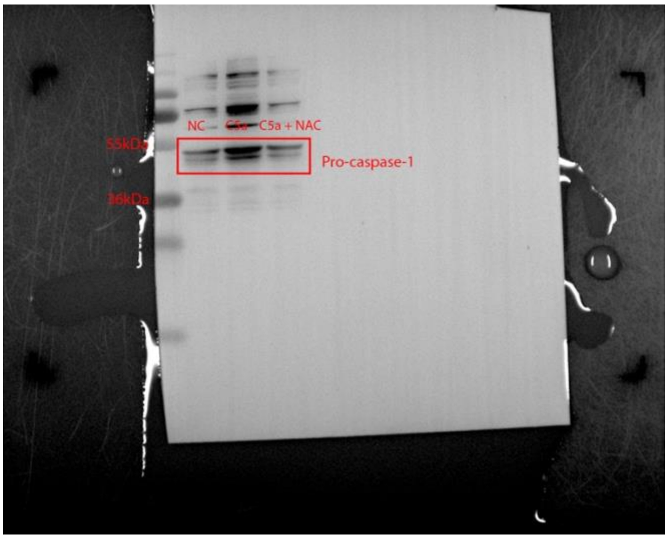

cleaved caspase-1

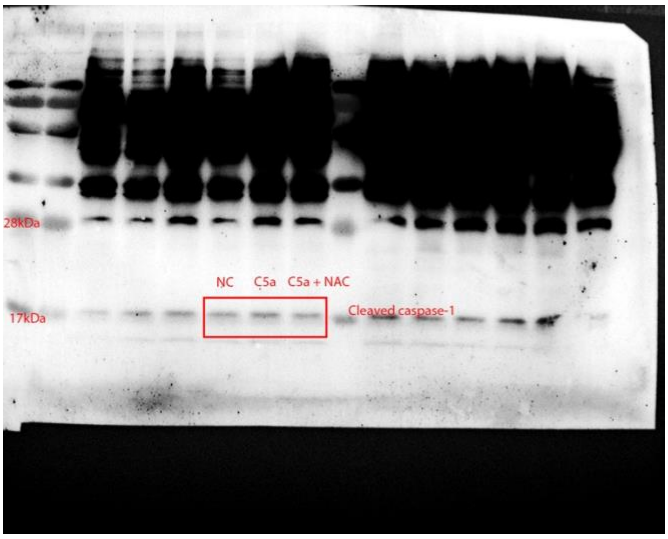

pro-IL-1β

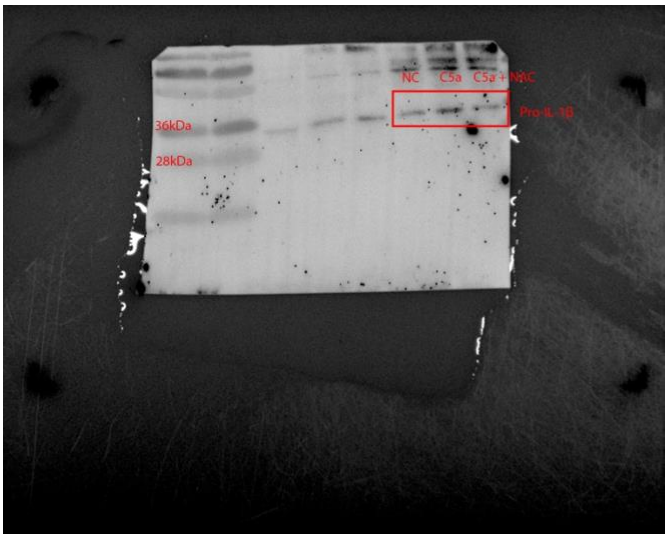

mature IL-1β

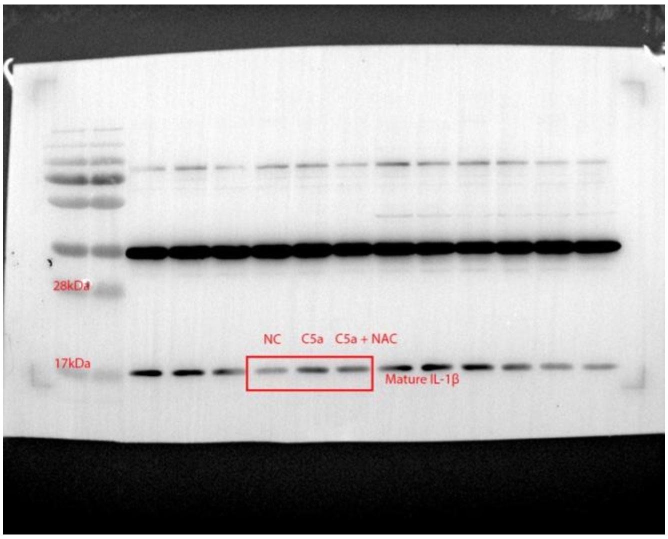

GAPDH

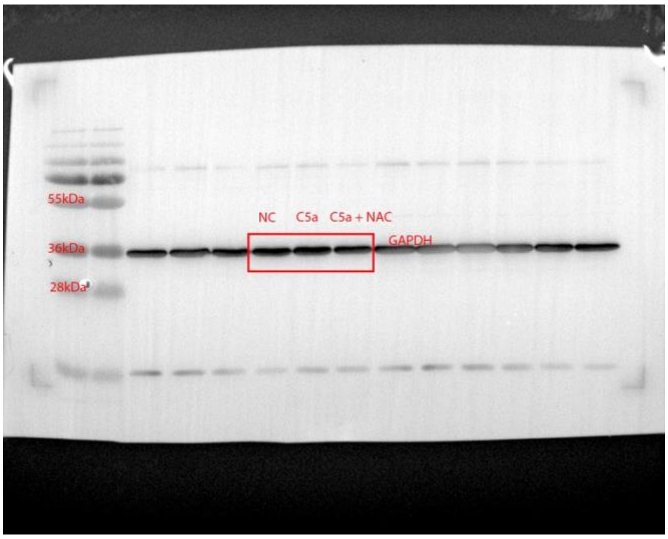

Figure 6

pro-caspase-1

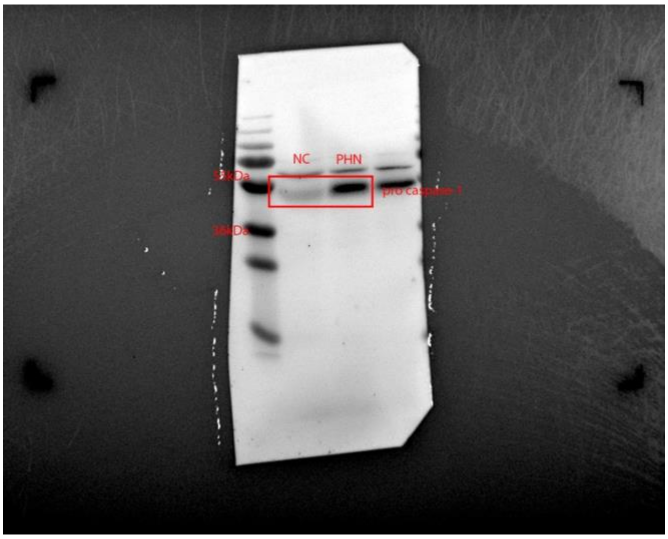

cleaved caspase-1

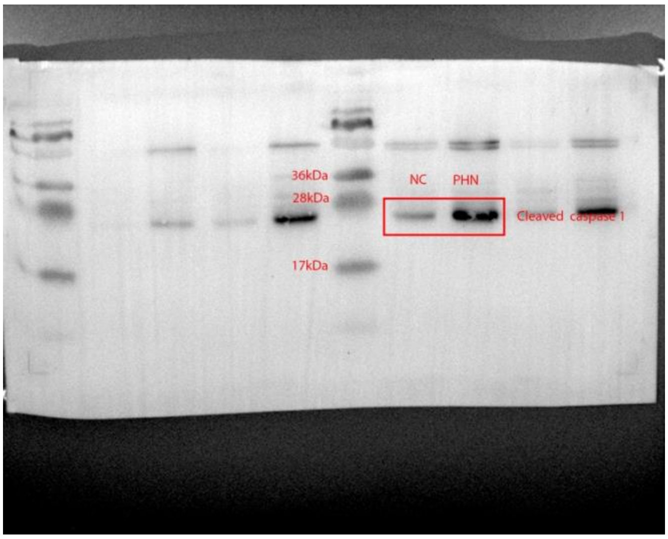

pro-IL-1β

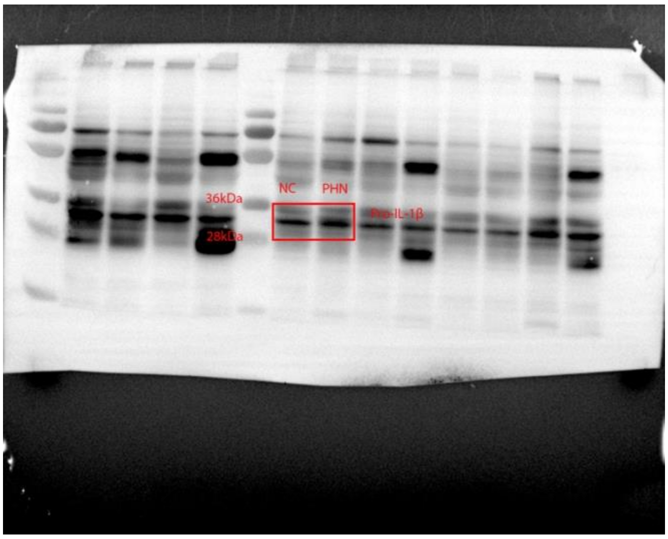

mature IL-1β

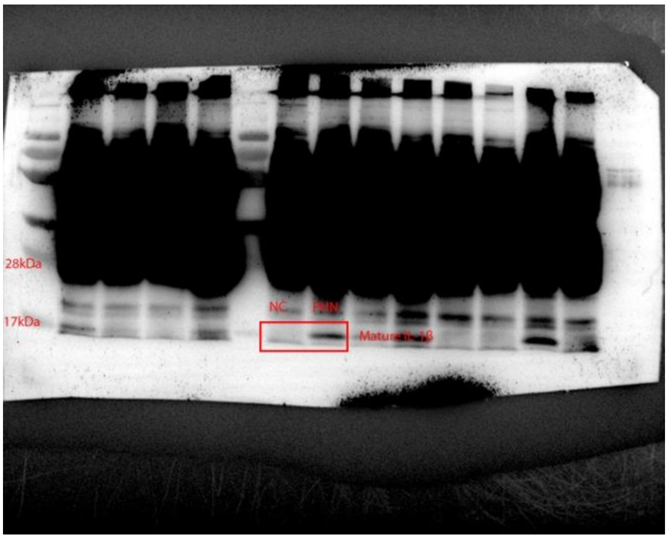

GSDMD-N

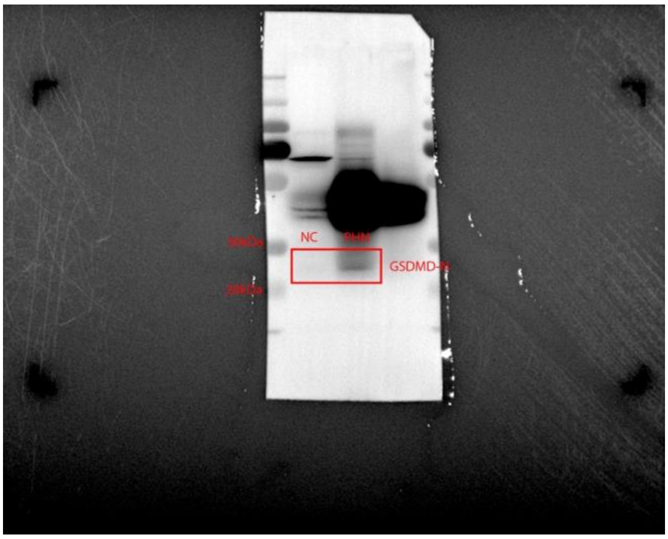

ASC

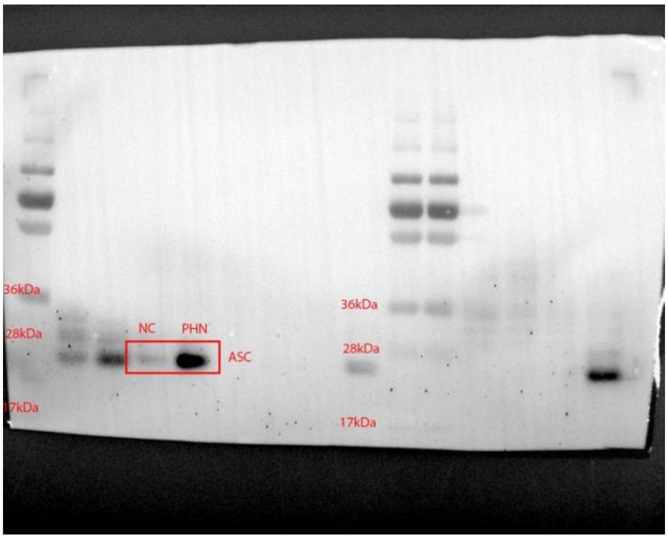

NLRP3

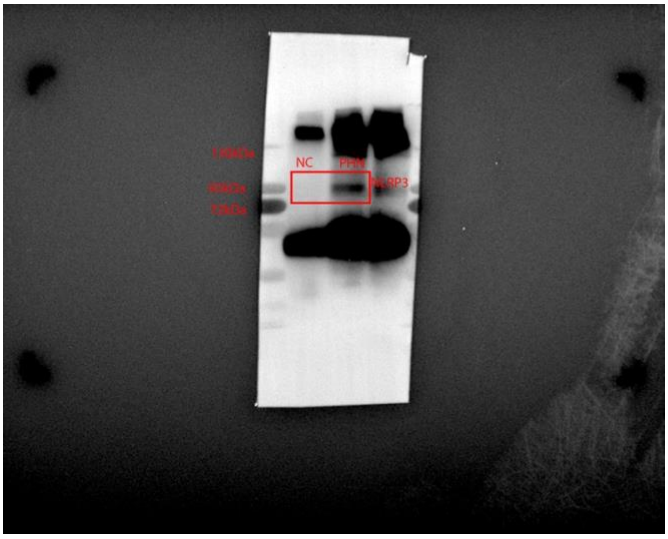

GAPDH

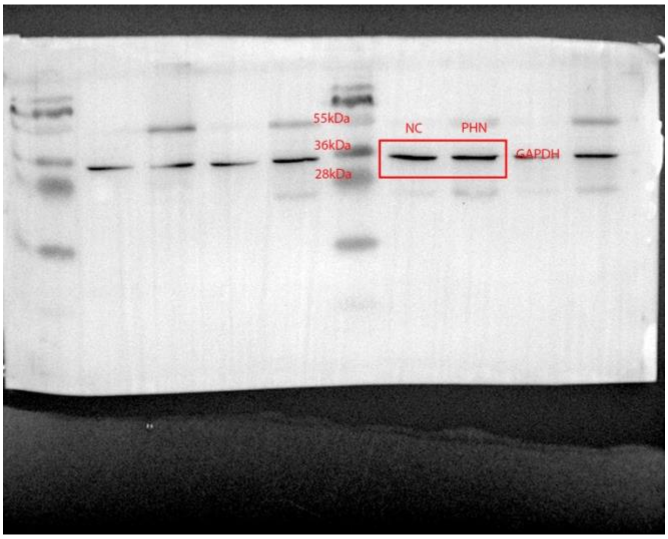

left of Figure 7B

NLRP3

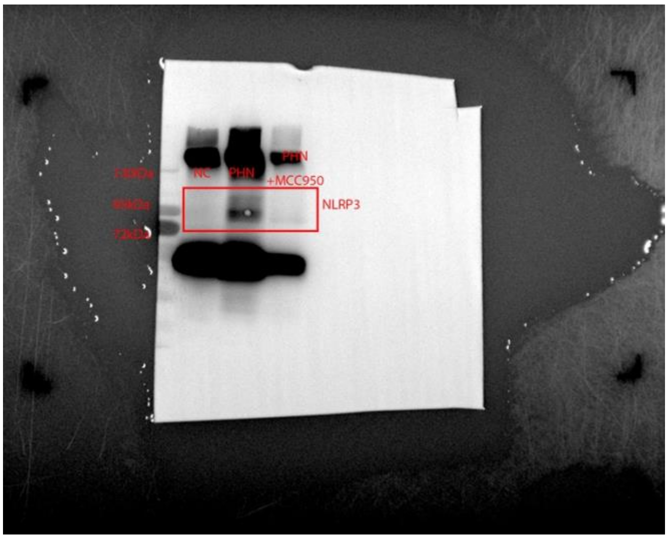

GAPDH

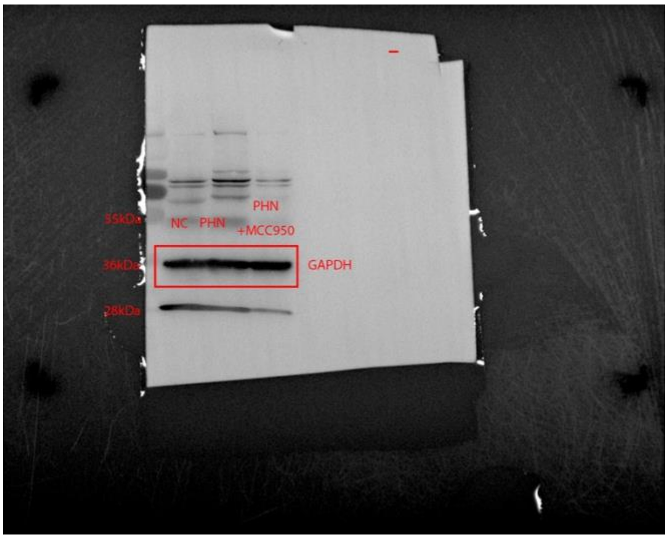

middle of Figure 7B

pro-caspase-1

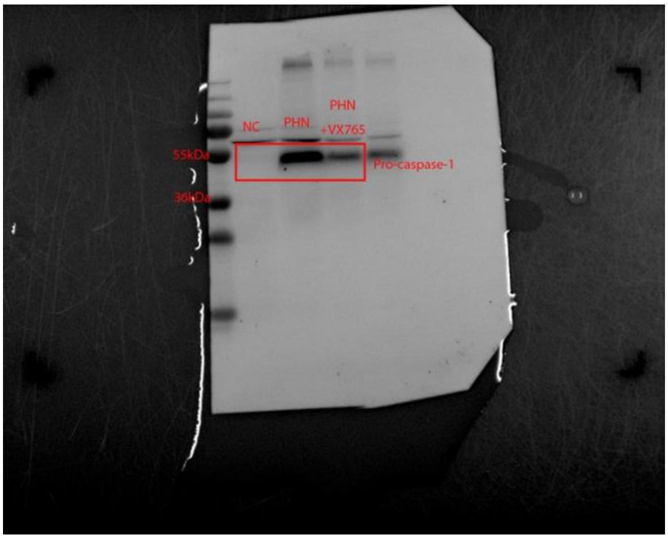

cleaved caspase-1

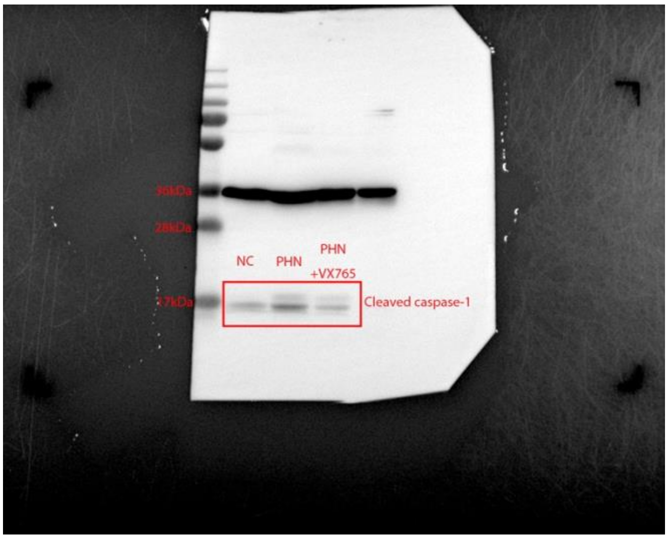

GAPDH

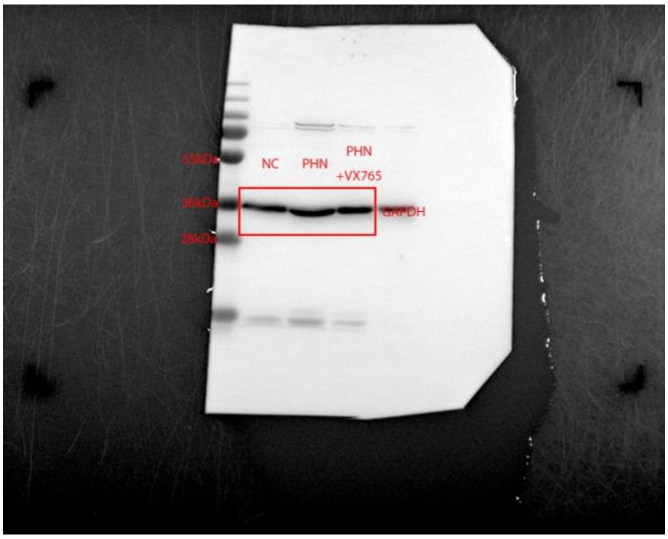

right of Figure 7B

GSDMD-N

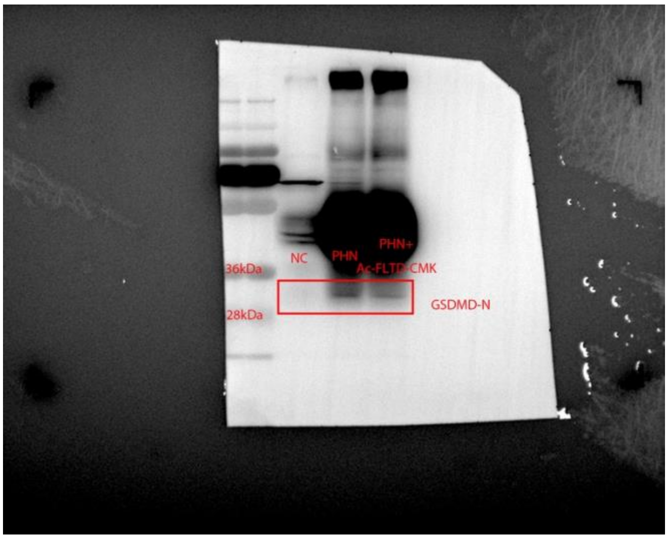

GAPDH

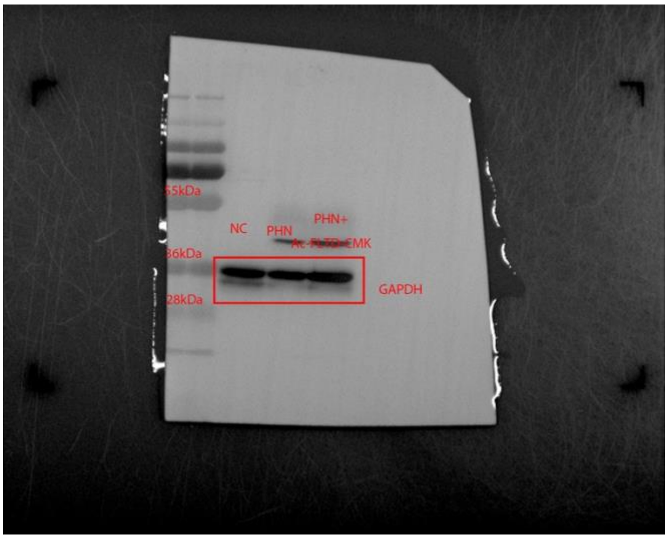

left of Supplementary Figure 2

NLRP3

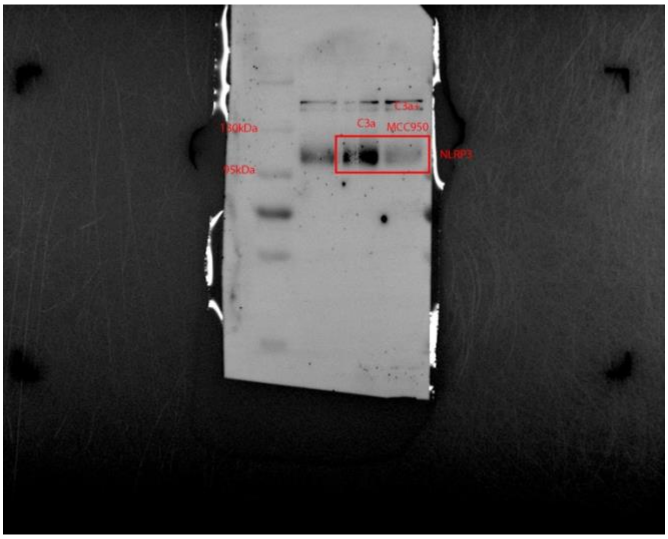

GAPDH

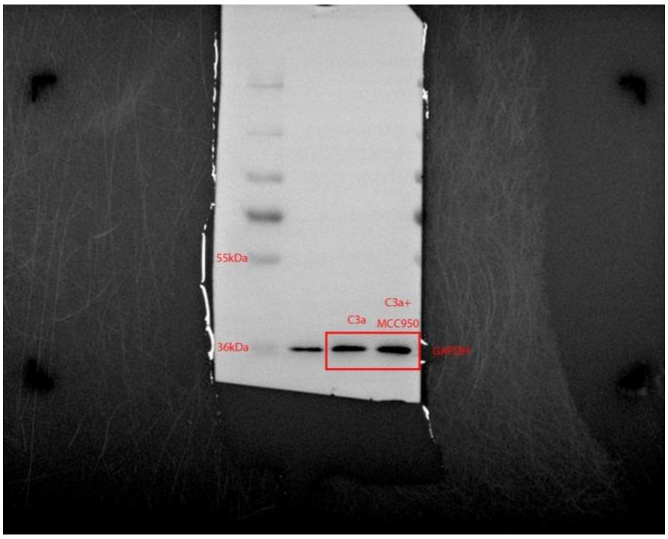

middle of Supplementary Figure 2

pro-caspase-1

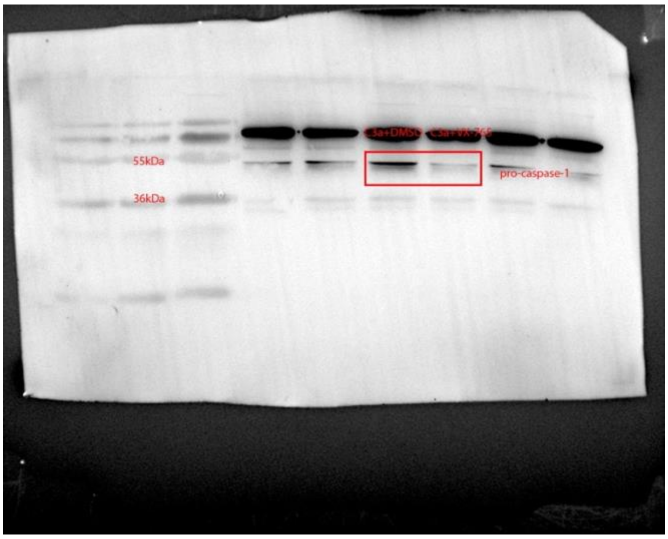

GAPDH

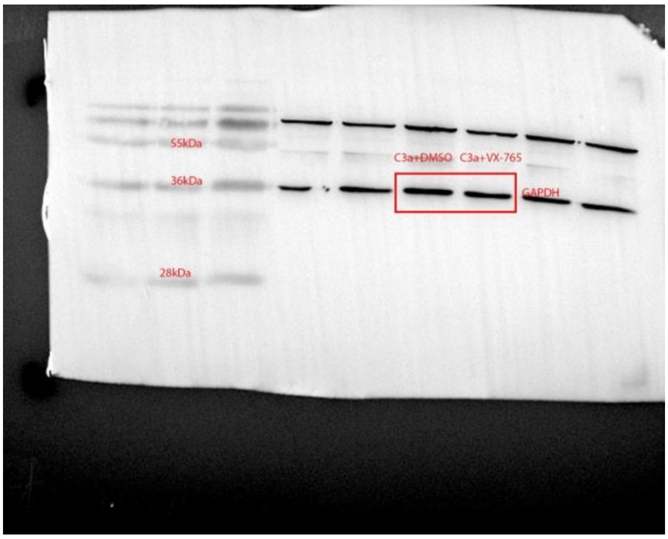

right of Supplementary Figure 2

GSDMD-N

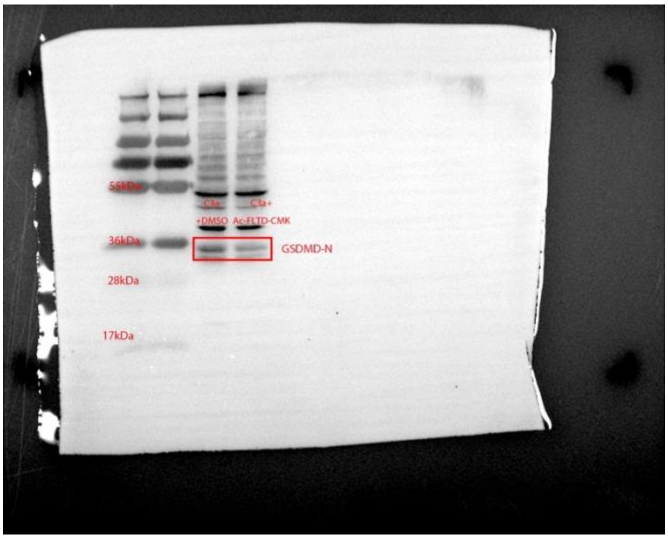

GAPDH

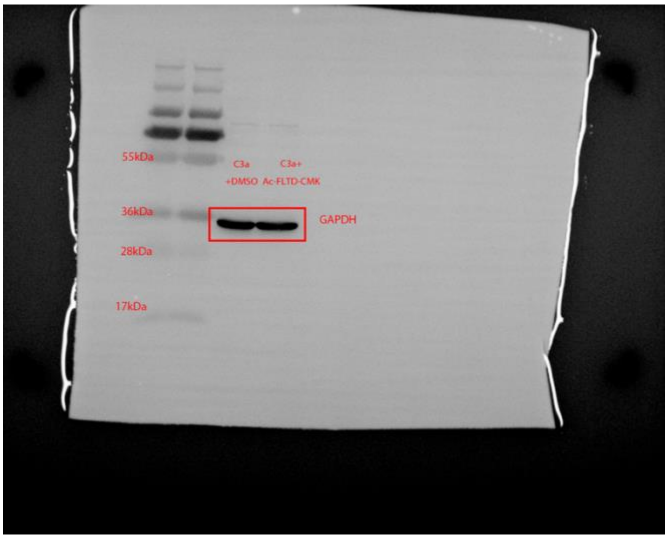

Supplement: Supplementary file 5 — Full scans of uncropped blots [file 41419_2022_4737_MOESM5_ESM.pdf]
